# Differential roles of cyclin–CDK1 complexes in cell migration and invasion

**DOI:** 10.1242/jcs.263697

**Published:** 2025-07-14

**Authors:** Joseph H. R. Hetmanski, Michael J. Jones, Matthew Hartshorn, Patrick T. Caswell, Matthew C. Jones

**Affiliations:** ^1^ Centre for Genome Engineering and Maintenance, Division of Biosciences, Dept. of Life Sciences, Brunel University of London, London UB8 3PH, UK; ^2^ Manchester Centre for Cell-Matrix Research, School of Biological Sciences, Faculty of Biology Medicine and Health, Manchester Academic Health Science Centre, The University of Manchester, Manchester M13 9PT, UK; ^3^ University of Plymouth, Peninsula Medical School, Plymouth PL6 8BU, UK

**Keywords:** CDK1, Cyclin, Migration, Invasion, 3D martix, RhoA

## Abstract

We have previously described a central role for CDK1 at the nexus of adhesion signalling and cell cycle progression, demonstrating that CDK1 has a non-canonical role in regulating integrin adhesion complexes and in the migration of cancer cells in 3D interstitial matrix. Here, we show that the CDK1-binding partners cyclinB1 and cyclinA2 also have roles in cell migration and invasion in both cancer and non-transformed cells. CyclinB1 plays a key role in RhoA activation to promote rear retraction in a membrane tension-dependent manner, whereas cyclinA2 has a general role in promoting motility. Knockdown of either cyclin significantly perturbs migration with contrasting phenotypes, whereas knockdown of both together has an additive effect, which arrests both migration and division. Our findings therefore describe how cyclin–CDK1 complexes orchestrate migration as well as division of cells, and that cyclinA2–CDK1 and cyclinB1–CDK1 complexes play distinct roles in motility.

## Teaser

**Highlighted Article:** Cyclin–CDK1 complexes have a previously undiscovered role in regulating normal and cancer cell migration in 3D matrices.

## INTRODUCTION

Cell migration is a fundamental process that is required for development and physiological processes, such as wound healing, angiogenesis and during immune responses (Yamada and Sixt, 2019). Furthermore, invasive cell migration into surrounding tissues is a hallmark of advanced cancer and contributes to metastasis in a variety of settings (Hanahan and Weinberg, 2011; Polacheck et al., 2013). Cell migration requires a concerted regulation of extracellular matrix adhesion, cytoskeletal dynamics and membrane tension that facilitate motility (Seetharaman and Etienne-Manneville, 2020; SenGupta et al., 2021). Enhancing our understanding of the molecular processes that coordinate migration is required to develop an integrated model of how cell migration is regulated in both physiological and pathological situations.

We have recently described a role for CDK1 in regulating integrin adhesion complexes and the actin cytoskeleton during cell cycle progression and migration (Hetmanski et al., 2021; Jones et al., 2018). This regulation occurs in part due to a direct interaction between CDK1 and the adhesion protein talin (Gough et al., 2021), via kinase-dependent regulation of the formin FMNL2 (Jones et al., 2018) and through RhoGTPase-mediated rear retraction (Hetmanski et al., 2021). Several other cytoskeletal targets for CDK1 have been identified, suggesting that regulation of the cytoskeleton is a crucial aspect of the role of CDK1 in cells and might represent the mechanism by which CDK1 controls both motility and division (Jones et al., 2019; Jones and Jones, 2024). In addition to our own studies in ovarian cancer cells migrating in 3D cell-derived matrices, knockdown of CDK1 abrogates cell migration and invasion in breast cancer cells (Wang et al., 2021), cholangiocarcinoma cells (Duan et al., 2021) and hepatocellular carcinoma (Dang et al., 2021), and inhibition of CDK1 activity reduces vimentin phosphorylation and migration in Schwann cells (Chang et al., 2012). This suggests that CDK1 plays a conserved role in regulating cell migration in both normal and cancer cells; however, the role for CDK1 in mediating cell migration in 2D and 3D environments remains poorly defined.

CDK1 function is primarily mediated through interaction with partner cyclin proteins that act to guide CDK1-specific phosphorylation (Morgan, 1995). Although many functions of CDK1 are associated with the cell cycle, cell cycle-independent roles have also been identified, including in DNA damage repair (Duda et al., 2016; Esashi et al., 2005; Hentges et al., 2014; Swaffer et al., 2016) and motility (Chang et al., 2012; Dang et al., 2021; Duan et al., 2021; Hetmanski et al., 2021; Wang et al., 2021). CDK1 forms complexes with A- and B-type cyclins (Malumbres, 2014), and regulation of adhesion complexes by CDK1 occurs primarily via association with cyclinA2 (Jones et al., 2018). CyclinA2 expression mediates cell migration, invasion and metastasis in hepatocellular (Fu et al., 2021), lung (Ruan et al., 2017) and breast carcinomas (Lu et al., 2022). However, low levels of cyclinA2 in prostate (Mashal et al., 1996), colorectal (Guo et al., 2021) and oral squamous cell carcinoma (Wang et al., 2008) are associated with increased invasiveness and more-aggressive disease. In normal mouse epithelial cells, loss of cyclinA2 drives epithelial-to-mesenchymal transition (EMT) and promotes mesenchymal cell migration (Bendris et al., 2014). These findings therefore describe a potential role for cyclinA2 in regulating CDK1 function during cell migration and invasion that is likely to be cell and context dependent. In contrast, little is known with regards to whether cyclinB1 can regulate cell motility, despite cyclinB1–CDK1 complexes being able to modify components of the cell migratory machinery, such as focal adhesions, actin, RhoA and microtubule dynamics during mitosis (Jones et al., 2019).

Our previous work has demonstrated that localisation of the RhoA guanine nucleotide exchange factor (GEF) Ect2 to caveolae at the rear of cells migrating in 3D matrices is dependent on CDK1 activity (Hetmanski et al., 2021). Low membrane tension at the cell rear promotes the formation of caveolae, which in turn drive RhoA activity via Ect2 and allow forward movement of the cell rear (Hetmanski et al., 2019). CDK1 is required for Ect2 activation during mitosis (Hara et al., 2006; Matthews et al., 2012; Niiya et al., 2006; Suzuki et al., 2015), and CDK1 inhibition also abrogates RhoA activity to prevent rear retraction (Hetmanski et al., 2021), indicating that there is significant overlap in the regulation of the cytoskeleton in division and migration. Because both cyclinA2 and cyclinB1 have been shown to regulate RhoA activity (Arsic et al., 2012; Matthews et al., 2012; Niiya et al., 2006), we here sought to determine which cyclin–CDK1 complexes were required for the modulation of cell migration in physiologically relevant 3D matrices. We demonstrate that cyclinB1 localises to the rear, whereas cyclinA2 shows a more diffuse localisation throughout the cytoplasm and nucleus of motile cells. Knockdown of either cyclinA2 or cyclinB1, or both simultaneously, perturbs cell motility in cell-derived matrices and invasion in collagen gels in a cell-cycle-independent manner in both normal and cancer cells. However, cyclinA2 and cyclinB1 depletion had differing effects on cell morphology, membrane tension, caveolae localisation and GTPase activity, suggesting that cyclin–CDK1 complexes have distinct roles in regulating cell migration and invasion with cyclinB1–CDK1 controlling rear retraction and cyclinA2–CDK1 important for protrusion. These findings therefore describe how changes in expression levels of cyclinA2, cyclinB1 and CDK1, or dysregulation of cyclin–CDK1 complexes, might impact upon cancer cell invasive migration in addition to proliferation and suggest that targeting CDK1 activity in invasive tumours, such as muscle invasive bladder cancer could be explored as a therapeutic option.

## RESULTS

### CyclinA2 and cyclinB1 control different aspects of cell migration in 3D cell-derived matrix

**Fig. 1.**
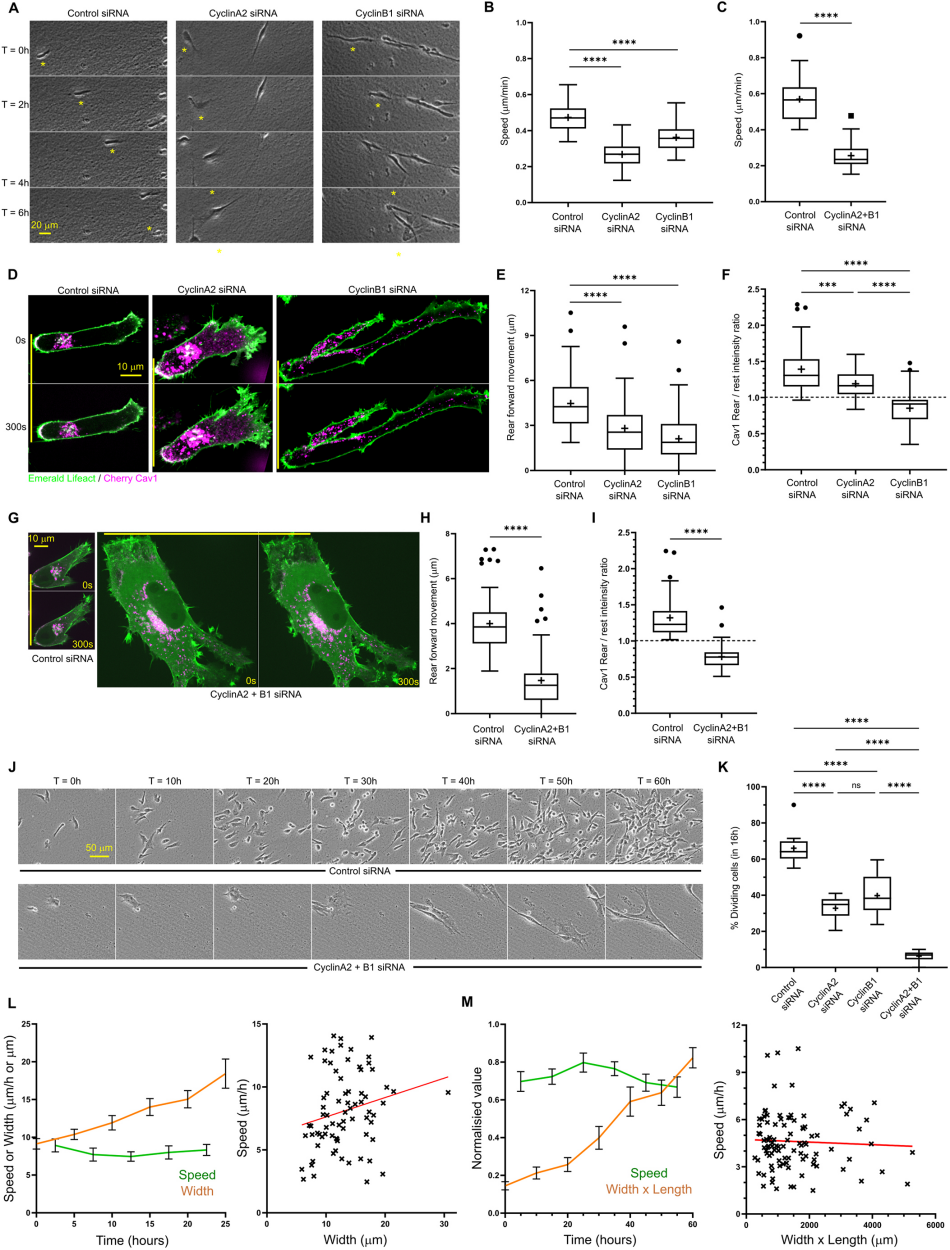
**CyclinA2 and cyclinB1 differentially regulate cell migration in 3D cell-derived matrix.** (A) Control, cyclinA2 and cyclinB1 knockdown A2780 ovarian cancer cells seeded in 3D CDM migrating over 6 h. The yellow asterisk denotes the position of the same cell at each time point. (B) Average migration speed of individual control, cyclinA2 and cyclinB1 siRNA A2780 cells in CDM across 16 h time-lapse. *n*=75 cells per condition analysed across three repeats. (C) Average migration speed of control and double cyclinA2+B1 siRNA A2780 cells in CDM across the first 16 h of time-lapse. *n*=75 cells per condition analysed across three repeats. (D) Control, cyclinA2 and cyclinB1 knockdown A2780 cells expressing Emerald–Lifeact (green) and mCherry–Cav1 (magenta) seeded in CDM; solid yellow line indicates initial rear position to compare to position after 300 s; cell moving from left to right (rear on the left, leading edge at the front) in this image and throughout. (E) Average forward rear movement of A2780 cells in CDM across 300 s time-lapse. *n*>40 cells analysed per condition across three repeats. (F) Ratio of mCherry–Cav1 intensity in the rearmost 20 pixels region/the rest of the cell for control, cyclinA2 and cyclinB1 siRNA cells. *n*=50 cells analysed per condition across three repeats. (G) Control and cyclinA2+B1 concomitant knockdown A2780 cells expressing Emerald–Lifeact (green) and Cherry Cav1 (magenta) seeded in CDM, yellow line denotes initial rear position for comparison purposes, same scale for both images. (H) Average forward rear movement of A2780 cells in CDM across 300 s time-lapse. *n*>40 cells analysed per condition across three repeats. (I) Ratio of mCherry–Cav1 intensity in the rearmost 20 pixels region/the rest of the cell for control and cyclinA2+B1 siRNA cells. *n*>48 cells analysed per condition across three repeats. (J) Time-lapse images of Control and cyclinA2+cyclinB1 concomitant knockdown A2780 cells seeded in CDM over 60 h, same position for each frame and same scale for each condition. (K) Percentage of control, cyclinA2, cyclinB1 and cyclinA2+B1 siRNA A2780 cells dividing during 16 h time period. *n*=12 positions analysed per condition across three repeats. (L) Left, direct comparison of average cell speed (green line) and cell width (orange line) over 25 h time period for enlarging cyclinA2 siRNA A2780 cells. Error bars show s.e.m. Right, average speed (*y*-axis) plotted against average width (*x*-axis) in 5 h time increments for cyclinA2 siRNA A2780 cells during 25 h time period; red line shows simple linear regression fit with non-significantly different non zero slope (0.154). 15 cells were analysed for both graphs (three independent experiments). (M) Left, direct comparison of average cell speed (green line) and cell width×cell length (orange line) over 60 h time period for enlarging cyclinA2+B1 siRNA A2780 cells, error bars show s.e.m. Right, average speed (*y*-axis) plotted against average width (*x*-axis) in 10 h time increments for cyclinA2+B1 siRNA A2780 cells during 60 h time period; red line shows simple linear regression fit with non-significantly different non-zero slope (−0.00004475). 15 cells were analysed for both graphs (three independent experiments). ****P<0.0001; ***P<0.001; ns, not significant (*P*>0.05) [ordinary one-way ANOVA with Tukey's multiple comparison test compared to control (B,E F and K); unpaired two-tailed Student's *t*-test (C,H,I)]. In box plots, the box represents the 25–75th percentiles, and the median is indicated by the line and mean by the +. The whiskers show the furthest data points that are not considered outliers (1.5 times the interquartile range away from the box).

We have previously shown that CDK1 is involved in cell migration in 3D environments and functions upstream of RhoA activity and contractile machinery at the rear of the cell (Hetmanski et al., 2021). We therefore investigated the role of the well-described CDK1-binding partners cyclinB1 and cyclinA2 in migrating A2780 ovarian cancer cells. Depletion of either cyclinA2 or cyclinB1, or both simultaneously, using two distinct siRNAs (Fig. S1A) significantly reduced the speed of A2780 ovarian cancer cell migration in 3D cell-derived matrices (CDMs) (Fig. 1A–C; Fig. S1D, Movies 1 and 2). Whereas cyclinB1-depleted cells were characterised by a longer and narrower shape consistent with a rear retraction defect (Cramer, 2013; Hetmanski et al., 2019) (Fig. 1A; Movie 1), the effect of cyclinA2 depletion was strikingly different, with cells displaying a wider and more rounded morphology. We therefore tested rear retraction directly in cells expressing mCherry–Cav1 to indicate caveolae localisation to the rear – a key component of the rear retraction positive feedback machinery we previously identified (Hetmanski et al., 2019). Whereas cyclinA2 and cyclinB1 knockdown, or both simultaneously, decreased rear movement compared to control siRNA (Fig. 1E,H), cyclinB1 depletion caused a more pronounced reduction, and cells displayed a greater loss of rear Cav1 localisation, whereas Cav1 remained localised at the rear of cyclinA2-depleted cells (Fig. 1D,F). This suggests that cyclinB1 perturbation has a direct effect on rear contractile machinery, whereas cyclinA2 knockdown reduces rear retraction, perhaps due to an overall decrease in cell speed. CyclinB1 knockdown increased the distance from the retracting rear to the nucleus, whereas cyclinA2 reduced this (Fig. S1C), further indicating that cyclinB1 is required for active translocation of the rear whereas cyclinA2 might influence the position of the nucleus by controlling other aspects of migration, such as protrusion. The CDK1–cyclinB effect on rear retraction and migration occurs primarily via cyclinB1, as cyclinB2 siRNA had no significant effect on migration speed whereas cyclinB1 plus cyclinB2 siRNA together had a similar effect on reducing migration speeds as cyclinB1 alone (Fig. S1B). Double cyclinA2 plus B1 (cyclinA2+B1) knockdown cells displayed hallmarks of the phenotypes seen when cyclinA2 or cyclin B1 were depleted individually – caveolae at the rear were lost and rear forward movement suppressed, similar to what was seen for individual cyclinB1 knockdown, but cells also showed wider protrusions, reminiscent of what was seen for individual cyclinA2 knockdown (Fig. 1G–I). Double knockdown of cyclinA2+B1 in cells caused a striking phenotype, whereby cells were not only unable to divide over 16 or even 60 h time-lapse acquisitions (Fig. 1J,K), but also had significantly reduced migration during those time frames while cells continued to increase in size (Fig. 1J,L; Movie 2). Interestingly, neither the increase in size observed with the double knockdown of cyclinA2+B1 nor the lesser increase in size with cyclinA2 knockdown correlated with a reduction in migration speed (Fig. 1L,M; Fig. S1E,F), even when cyclinA2 cells were <10 µm wide (near the start of the long-term acquisitions) and therefore comparable in width to control cells . This suggests that perturbed migration does not occur as a result of cell size alteration preventing migration through CDMs.

Taken together, these data show that both cyclinA2 and cyclinB1 perturbation have severe effects on cell migration in different ways, whereas concomitant perturbation of both leads to a striking loss of migratory as well as division capacity associated with cell size increase.

### CyclinA2 and cyclinB1 differentially regulate cell morphology and RhoA activity in cells within 3D collagen hydrogels

**Fig. 2.**
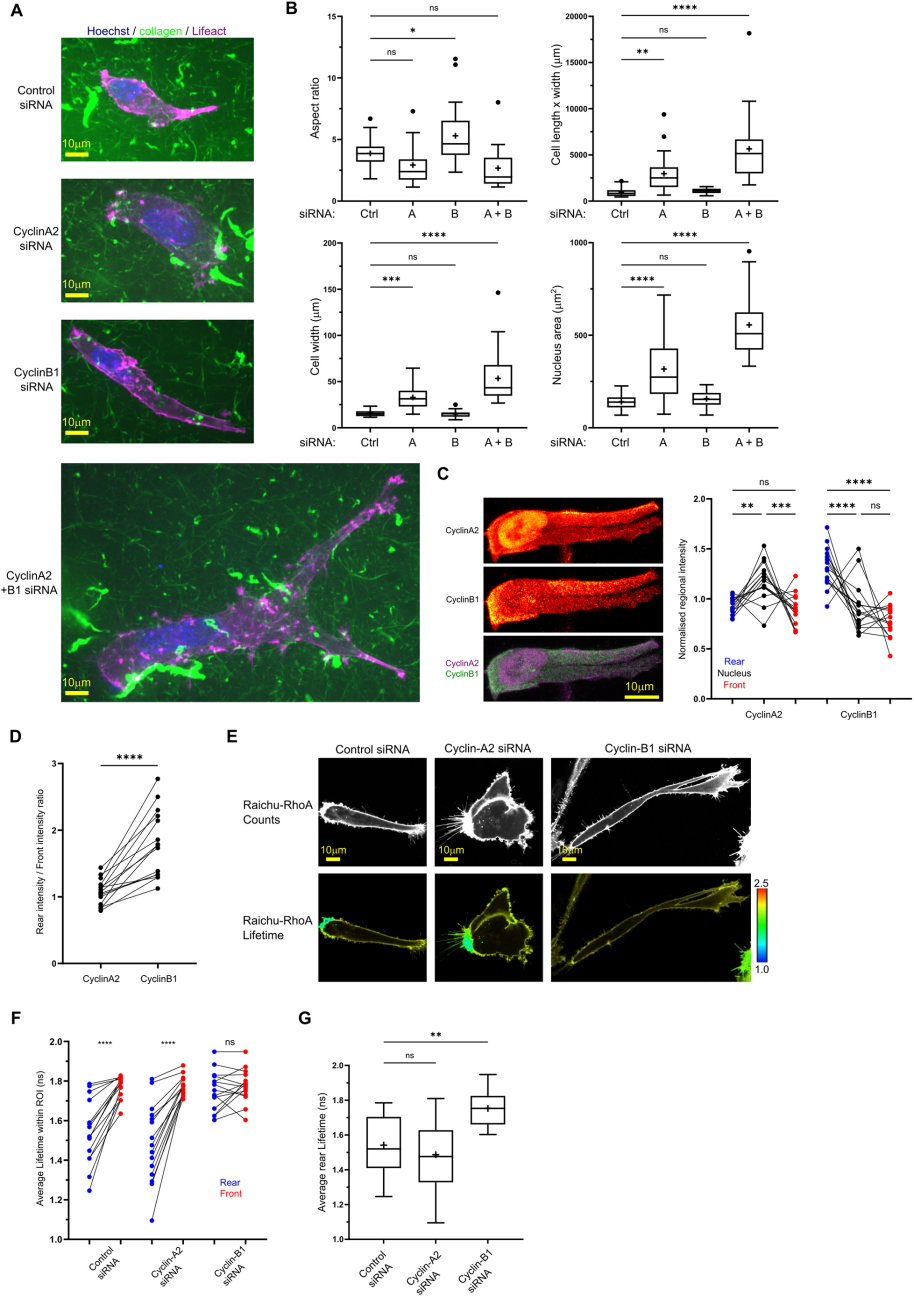
**CyclinA2 and cyclinB1 depletion have distinct effects on cell and RhoA activity.** (A) Control, cyclinA2, cyclinB1 and cyclinA2+B1 concomitant knockdown A2780 cells stably expressing iRFP670–Lifeact and dyed with Hoechst seeded directly into 3D soft (∼1.5 mg/ml) collagen, where 1/10 collagen fibres are labelled with GFP, same scale used for all conditions. (B) Quantification of cell size and shape metrics of control (Ctrl), cyclinA2 (denoted A), cyclinB1 (denoted B) and cyclinA2+B1 (A+B) siRNA cells. All measurements of length and width taken at widest and longest positions. Aspect ratio (top left) calculated as maximum cell length divided by maximum cell width. *n*>25 cells analysed per condition across three repeats; the same cells were analysed for all four metrics/graphs. (C) Left, fixed A2780 cell seeded in CDM immuno-stained for cyclinA2 (top, magenta in composite image) and cyclinB1 (middle, green in composite image), red hot LUT applied. Right, normalised cyclinA2 and cyclinB1 intensities in rear (blue), nuclear (black) and front (red) regions (regions of the same cell joined by black line). *n*=12 cells analysed across three repeats. (D) Rear/front region cyclinA2 and cyclinB1 intensity ratios (cyclinA2 and cyclinB1 ratios for the same cell joined by black line). *n*=12 cells analysed as in C. (E) Control, cyclinA2 and cyclinB1 knockdown A2780 cells seeded in CDM transfected with a GFP–RFP Raichu–RhoA probe. Counts (top) and lifetime (bottom) of donor GFP channel as per the colour code range (numbers in the colour scale represent time in ns). Blue denotes shorter lifetime (high activity); yellow–red denotes higher lifetime (low activity). (F) Average FLIM lifetime of Raichu–RhoA-expressing A2780 cells in manually drawn rear (blue) and front (red) ROIs (rear/front regions of the same cell joined by black line). *n*>15 cells analysed per condition across three repeats. Note the lower lifetime denotes higher RhoA activity. (G) Average lifetime in rear ROI (same A2780 cells as in F analysed). *n*>15 cells analysed per condition across three repeats. ****P<0.0001; ***P<0.001; **P<0.01; *P<0.05; ns, not significant (*P*>0.05) [ordinary one-way ANOVA with Tukey's multiple comparison test compared to control (B,G); paired two-tailed Student's *t*-tests (C,D,F)]. In box plots, the box represents the 25–75th percentiles, and the median is indicated by the line and mean by the +. The whiskers show the furthest data points that are not considered outliers (1.5 times the interquartile range away from the box).

Building on the differing phenotypic effects of cyclin knockdowns on A2780s in CDMs, which are a confined 3D environment (∼20 µm thick, such that cells are unable to move up and down in the *z* direction), we next tested whether such diverse responses were present in a fully 3D-matrix environment. We seeded control or cyclin siRNA iRFP670 Lifeact-expressing A2780s directly into soft collagen gels before analysing cell morphology after 16 h (Fig. 2A). Similar to what was seen in the CDM environment, cyclinB1-depleted cells appeared to be long and narrow, with a significantly increased aspect ratio (length-to-width) in comparison to control cells, whereas cyclinA2 siRNA cells were significantly wider, bigger overall (as defined by length×width) and had a larger nucleus. Again, an additive effect of both cyclinA2+B1 depletion was observed, whereby cells were considerable larger, longer and wider with larger nuclei than in control, cyclinA2- or cyclinB1-depleted cells (Fig. 2B). Consistent with observations in 2D, cyclinA2 was predominantly observed in the nucleus of cells cultured in 3D with a proportion of cyclinA2 also being localised to the rear and front of migrating cells (Fig. 2C). In contrast, cyclinB1 was predominantly cytoplasmic and displayed significant enrichment to the rear of migrating cells (Fig. 2C,D).

We next explored whether the different morphologies and phenotypes observed corresponded with different RhoA GTPase activity. In CDM, we found that cyclinA2 knockdown had little effect on RhoA polarisation or activity at the rear, whereas cyclinB1 knockdown significantly decreased RhoA activity as measured by an increase in the fluorescence lifetime of the Raichu–RhoA probe specifically at the rear, leading to an overall loss of front-rear RhoA polarity (Fig. 2E–G). CyclinA2+B1 siRNA together also abrogated front-rear RhoA polarity via a decrease of RhoA activity at the rear (Fig. S2A). This further supports the hypothesis that cyclinB1 has a specific role upstream of RhoA and contractile machinery at the rear, whereas cyclinA2 affects cell morphology and migration via an alternative mechanism, potentially through the regulation of integrin adhesion complexes and talin function in cell protrusions.

### CyclinB1 both responds to and affects membrane tension at the rear

**Fig. 3.**
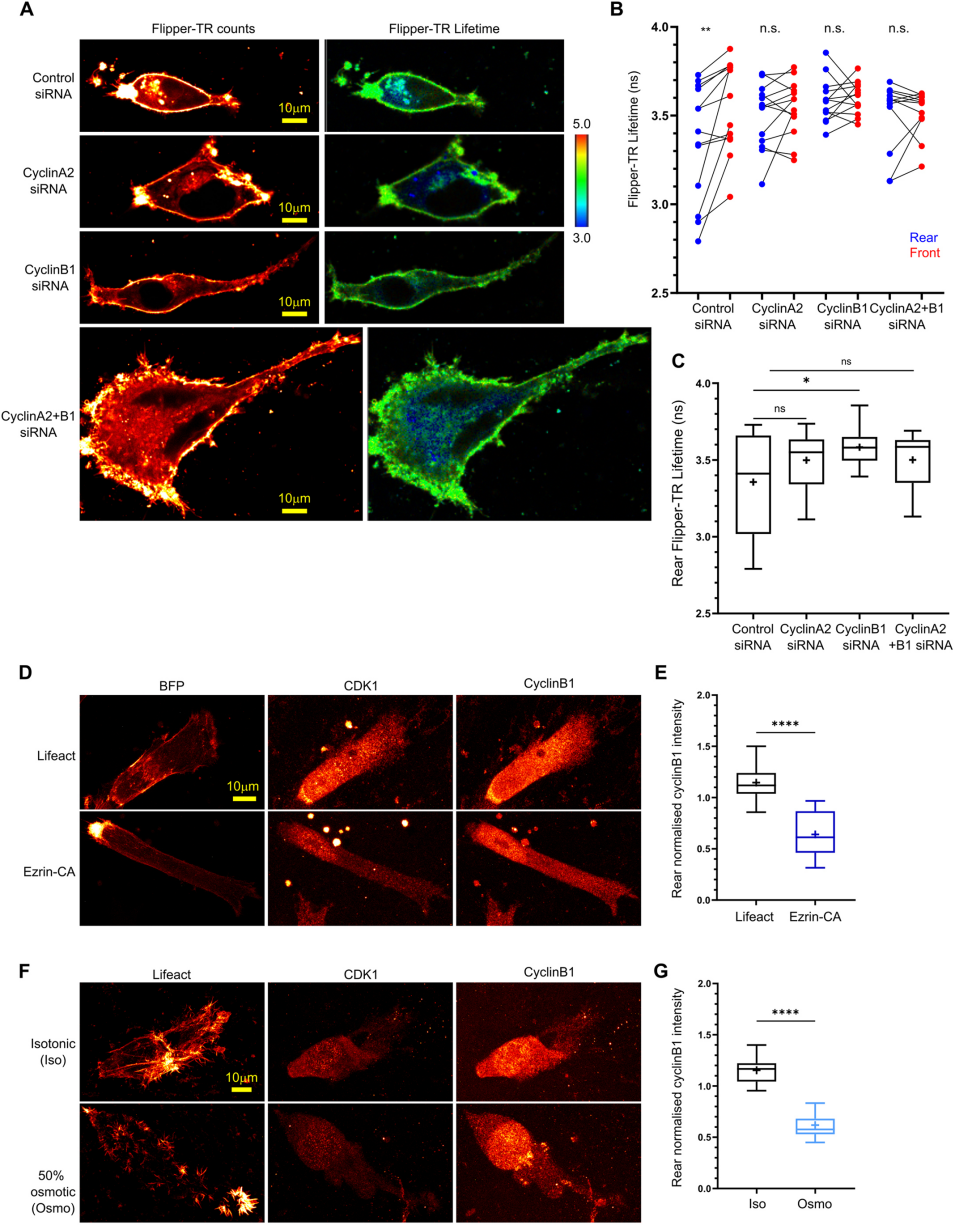
**CyclinB1 maintains low membrane tension at the cell rear in migrating cells.** (A) Control, cyclinA2, cyclinB1 and cyclinA2+B1 concomitant knockdown A2780 cells seeded directly into soft collagen and dyed with membrane tension probe FlipperTR. Counts (left, ‘red hot’ LUT applied) and lifetime (colour coded lifetime range are shown; numbers in the colour scale represent time in ns). Blue–green denote shorter lifetime and lower tension; yellow–red denote longer lifetime and higher tension for of 488 nm excitation and 575–625 nm emission. (B) Average Flipper-TR lifetime of manually drawn rear (blue) and front (red) ROIs in A2780 cells in collagen gels (rear/front regions of the same cell joined by black line). *n*>10 cells analysed per condition across three repeats. (C) Average Flipper-TR lifetime in rear ROI of xontrol, cyclinA2 (A), cyclinB1 and cyclinA2+B1 siRNA cells (same A2780 cells as in B analysed). *n*>10 cells analysed per condition across three repeats. (D) Fixed A2780 cells seeded in CDM transfected with BFP–Lifeact (top) or BFP–Ezrin-CA (bottom; constitutively active, to increase membrane tension) and immuno-stained with CDK1 and cyclinB1. A ‘red hot’ LUT has been applied. (E) Normalised average cyclinB2 staining intensity at the rear in cells transfected with BFP–Lifeact or Ezrin-CA. *n*>15 cells analysed per condition across three repeats. (F) Fixed A2780 cells seeded in CDM in normal isotonic medium (top) or subjected to osmotic shock by addition of 50% water for 30 min prior to fixation (bottom, to increase membrane tension) and immuno-stained for CDK1 and cyclinB1. A ‘red hot’ LUT has been applied. (G) Normalised average cyclinB2 staining intensity at the rear in cells in normal (Iso) or 50% water (Osmo) media. *n*>15 cells analysed per condition across three repeats. ****P<0.0001; **P<0.01; *P<0.05; ns, not significant (*P*>0.05) [paired two-tailed Student's *t*-tests (B); unpaired two-tailed Student *t*-tests (E,G); ordinary one-way ANOVA with Tukey's multiple comparison test compared to control (C)]. In box plots, the box represents the 25–75th percentiles, and the median is indicated by the line and mean by the +. The whiskers show the furthest data points that are not considered outliers (1.5 times the interquartile range away from the box).

We have found previously that membrane tension plays a key role in driving rear retraction as part of a positive feedback loop (Hetmanski et al., 2019). We therefore tested how cyclin perturbation affected membrane tension using fluorescence lifetime imaging microscopy (FLIM) of the FlipperTR (Colom et al., 2018; Pandzic et al., 2022) in 3D collagen hydrogels. As we found previously in CDM (Hetmanski et al., 2019), A2780 cells displayed lower FlipperTR lifetime at the rear than at the front, indicating lower tension at the rear (Fig. 3A,B). Perturbation of either cyclinA2 or cyclinB1 individually or both together all resulted in a loss of this membrane tension differential (Fig. 3A,B); however, only cyclinB1 siRNA specifically increased membrane tension at the rear compared to that seen in control cells (Fig. 3C). This suggests that cyclinB1 exerts a more direct effect on membrane tension at the rear, whereas cyclinA2 or cyclinA2+B1 affect membrane tension polarity by altering tension of the membrane more globally via changes in cell shape. or indirectly via the overall decrease in motility which breaks the membrane tension–contractility positive feedback loop.

Our data so far indicate a specific role for CDK1–cyclinB1 in regulating membrane tension and RhoA activity at the retracting rear (Figs 1 and 2 ; Hetmanski et al., 2021). Therefore, we next wanted to ascertain whether the endogenous localisation of CDK1 and cyclinB1 was dependent on membrane tension. Migrating A2780s in CDM displayed localisation of both CDK1 and cyclinB1 at the rear (Fig. 3D,F; Fig. S3A,B) when cells were unperturbed or expressing BFP–Lifeact in normal isotonic medium. When membrane tension was globally increased either by expression of constitutively active (CA) BFP–Ezrin or osmotic shock with 50% water, the rear localisation of cyclinB1 and CDK1 was lost (Fig. 3D–G), and instead these proteins were both confined more to the nuclear compartment. CDK1 localisation was also more confined to the nucleus when cells were treated with individual cyclinB1 or A2 siRNAs or both together (Fig. S3A,B). These data indicate that rear membrane CDK1 localisation is dependent on the availability of cyclin-binding partners and that, in conditions where this is perturbed, CDK1 is retained in the nucleus. Moreover, these data suggest that CDK1–cyclinB1 signalling at the rear is both dependent on membrane tension and directly affects membrane tension, indicating that CDK1–cyclinB1 is involved in the rear retraction positive feedback loop.

### CyclinA2 and B1 are required for migration of non-cancer RPE cells

**Fig. 4.**
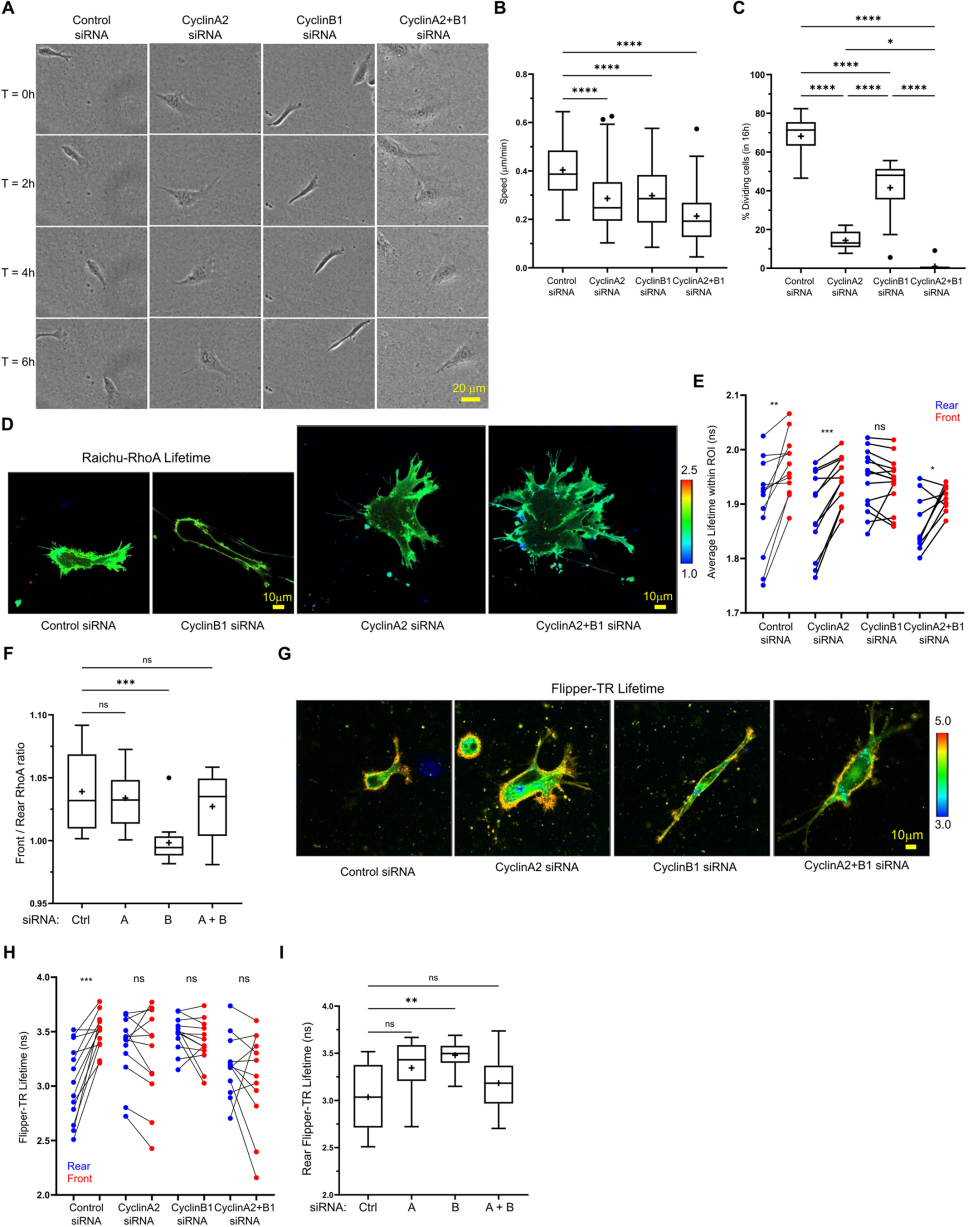
**Cyclin perturbation in non-cancer RPE cells demonstrates conserved differential roles for cyclinA2 and B1 in invasive migration.** (A) Control, cyclinA2, cyclinB1 and cyclinA2+B1 concomitant knockdown RPE cells seeded in CDM migrating over 6 h. The same cell moving from left to right is shown in each frame. (B) Average migration speed of RPE cells in CDM across 16 h time-lapse. *n*=75 cells per condition analysed across three repeats. (C) Percentage of control, cyclinA2, cyclinB1 and cyclinA2+B1 siRNA RPE cells dividing during 16 h time period. *n*=12 positions analysed per condition across three repeats. (D) Control, cyclinA2, cyclinB1 and cyclinA2+B1 concomitant knockdown RPE cells seeded in collagen gels transfected with the GFP–RFP Raichu–RhoA probe. The lifetime of donor GFP channel as per the colour code range is shown (numbers in the colour scale represent time in ns). Blue denotes shorter lifetime (high activity); green–yellow denotes higher lifetime (low activity). (E) Average FLIM lifetime of Raichu–RhoA-expressing RPE cells in manually drawn rear (blue) and front (red) ROIs (rear/front regions of the same cell joined by black line), *n*>15 cells analysed per condition across three repeats. Note the lower lifetime denotes higher RhoA activity. (F) Average lifetime in rear ROI (same RPE cells as in C and F analysed). *n*>15 cells analysed per condition across three repeats. (G) Control, cyclinA2, cyclinB1 and cyclinA2+B1 concomitant knockdown RPE cells seeded directly into soft collagen and dyed with membrane tension probe FlipperTR. Lifetime of 488 excitation, 575–625 emission as per the colour code range is shown (numbers in the colour scale represent time in ns). Blue–green denotes shorter lifetime and lower tension; yellow–red denotes longer lifetime and higher tension). (H) Average Flipper-TR lifetime of manually drawn rear (blue) and front (red) regions of interest in RPE cells in collagen gels (rear/front regions of the same cell joined by black line). *n*>10 cells analysed per condition across three repeats. (I) Average Flipper-TR lifetime in rear ROI of Control (Ctrl), cyclinA2 (denoted A), cyclinB1 (denoted B) and cyclinA2+B1 (A+B) siRNA cells (same RPE cells as in B analysed). *n*>10 cells analysed per condition across three repeats. ****P<0.0001; ***P<0.001; **P<0.01; *P<0.05; ns, not significant (*P*>0.05) [paired two-tailed Student's *t*-tests (E,H); ordinary one way ANOVA with Tukey's multiple comparison test compared to control (B,C,F,I)]. In box plots, the box represents the 25–75th percentiles, and the median is indicated by the line and mean by the +. The whiskers show the furthest data points that are not considered outliers (1.5 times the interquartile range away from the box).

Having demonstrated that cyclinA2 and cyclinB1 regulate the motility of cancer cells, we next wanted to test whether these cyclins have conserved roles in regulation of cell migration, morphology, RhoA activity and membrane tension in a non-cancer cell line. Immortalised retinal pigment epithelial (RPE) cells are non-transformed and near diploid, move and divide freely, and have been used extensively in cell cycle studies (Barbiero et al., 2022; Trotter and Hagan, 2020). In RPE cells, knockdown of cyclinA2, cyclinB1 or both cyclinA2+B1 (Fig. S4A) all severely reduced migration and cell division in 3D CDM (Fig. 4A–C); in addition, cyclinB1-depleted cells were longer and thinner, cyclinA2-depleted cells were wider and larger overall, and cyclinA2+B1 knockdown together showed an additive effect where cells were largest, moved the least and failed to undergo division. Similar effects were seen in 3D collagen gels (Fig. 4C) with cyclinA2 or cyclinA2+B1 knockdown cells also displaying many more leading-edge protrusions, whereas cyclinB1 knockdown resulted in a loss of rear RhoA activity and cyclinA2+B1 siRNA led to a mis-localisation of RhoA activity (Fig. 4D–F). For RPE cells in 3D collagen, knockdown of either cyclin individually or both together resulted in the loss of front-rear membrane tension polarity whereas only cyclinB1 siRNA specifically increased membrane tension at the rear (Fig. 4G–I). Altogether, these data illustrate that the phenotypic consequences of cyclinA2 and cyclinB1 siRNA observed previously in ovarian cancer A2780s are conserved in non-cancer RPEs, whereby during motility, cyclinB1 is involved in rear retraction while cyclinA2 is involved elsewhere. Furthermore, analysis of cell migration before and after mitosis demonstrated that no significant change in cell speed occurred, indicating that RPE1 cells do not exhibit cell cycle phase-dependent changes in migration in CDMs (Fig. S4A,B). This suggests that the decrease in migration observed in cyclin knockdown cells is not due to modification of cell cycle progression but due to a specific role for cyclins in regulating the migration machinery as described.

### Cyclin–CDK1 complexes regulate invasion in ovarian and bladder cancer cells

**Fig. 5.**
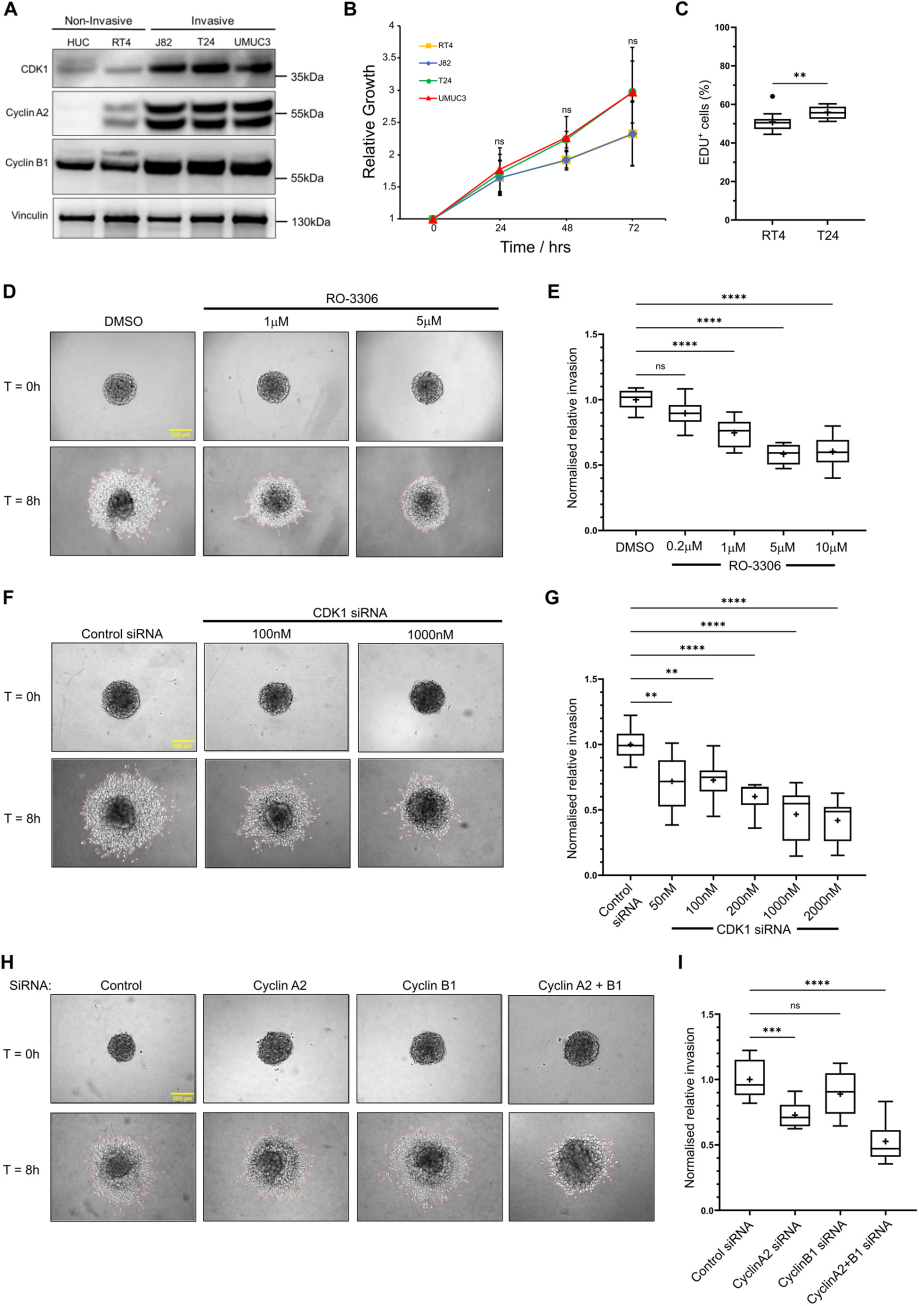
**Loss of CDK1 and cyclin activity perturbs T24 bladder cancer cell invasion in 3D spheroid models.** (A) Western blot showing differences in expression of CDK1 and its respective cyclins cyclinA2 and cyclinB1, between non-invasive and invasive bladder cell lines. Blot is representative of three repeats. (B) Quantification of growth curve analysis showing relative growth of bladder cell lines at 24 h intervals for a total of 72 h. Error bars show standard deviation (*n*=3). (C) Quantification of EDU proliferation assay comparing the percentage of EDU positive cells between non-invasive RT4 and invasive T24 bladder cells (*n*=3). (D) T24 spheroid invasion assay showing T24 spheroids seeded into 2.5 mg/ml collagen gels supplemented with DMSO or varying concentrations of RO-3306 at 0 h and 8 h timepoints. Representative images for only DMSO, 1 µM RO-3306 and 5 µM RO-3306 are shown. (E) Quantification of invasion area of RO-3306 treated T24 spheroids relative to DMSO treated spheroids. 9 spheroids per condition across three repeats was used for analysis. (F) T24 spheroid invasion assay showing T24 spheroids generated from CDK1 knockdown T24 cells, seeded into 2.5 mg/ml collagen gels at 0 h and 8 h timepoints. Representative images for only control siRNA, 100 nM CDK1 siRNA and 1000 nM CDK1 siRNA representative images are shown. (G) Quantification of invasion area of CDK1 knockdown spheroids relative to control siRNA treated spheroids. 9 spheroids per condition across three repeats was used for analysis. (H) T24 spheroid invasion assay showing T24 spheroids generated from cyclinA2, cyclinB1 or a combination of cyclinA2+B1 knockdown T24 cells, seeded into 2.5 mg/ml collagen gels at 0 h and 8 h timepoints. (I) Quantification of invasion area of cyclin-knockdown spheroids relative to control siRNA treated spheroids. 9 spheroids per condition across three repeats was used for analysis. ****P<0.0001; ***P<0.001; **P<0.01; ns, not significant (*P*>0.05) [one-way ANOVA and Tukey's post hoc test to compare between group means at 24, 48 and 72 h timepoints (B); unpaired two-tailed Student's *t*-test (C); ordinary one-way ANOVA with Tukey's multiple comparison test compared to control (E,G,I)]. In box plots, the box represents the 25–75th percentiles, and the median is indicated by the line and mean by the +. The whiskers show the furthest data points that are not considered outliers (1.5 times the interquartile range away from the box).

Given that CDK1–cyclin complexes control the 3D migration of normal and cancer cells, we next tested their role in long-range invasion through 3D collagen-I gels. Knockdown of CDK1 or cyclinA2+B1 together resulted in significantly lower invasion over 72 h in both soft (∼1.5 mg/ml) and stiff (∼5 mg/ml) collagen (Fig. S5A). We next sought to corroborate the role for cyclin–CDK1 complexes in mediating invasive migration in an alternative cancer type. Patients with bladder cancer (BC) tumours that have invaded the surrounding muscle have poor survival rates in comparison to non-muscle invasive tumours and limited treatment options, with the mechanisms that contribute to invasive migration by BC cells remining poorly defined (Smith et al., 2024). Initially, we compared expression levels of CDK1, cyclinA2 and cyclinB1 between primary bladder epithelial cells (HUCs), non-invasive BC cells (RT4; Fig. S5B) and invasive BC cells (J82, T24 and UMUC3). Expression of CDK1, cyclinA2 and cyclinB1 were all upregulated in the three invasive cell types compared to the two non-invasive lines as assessed by western blotting (Fig. 5A); however, this differential expression was not a consequence of drastically increased cell proliferation, as all cancer cells proliferated at similar rates (Fig. 5B,C). In addition, analysis of TCGA expression data via gene expression profiling interactive analysis (GEPIA) demonstrated that CDK1, cyclinA2 and cyclinB1 are observed at increased expression levels in bladder cancer tissues, whereas CDK2, CDK4 and cyclinD1 are not differentially expressed (Fig. S5G). We took forward the T24 cells for further analysis as they show enhanced levels of invasion (Fig. S5B), and demonstrated that they too show rear localised caveolae, rapid rear forward movement (Fig. S5C) and high RhoA activity localised specifically to the rear (Fig. S5D). In spheroid invasion assays, T24 cells displayed a dose-dependent reduction in invasive capacity through 2.5 mg/ml collagen-I gels in response to CDK1 inhibition with RO-3306 up to 5 µM (Fig. 5D). T24 cell invasion was also reduced in a dose-dependent manner following siRNA-mediated knockdown of CDK1 (Fig. 5E) and upon knockdown of either cyclinA2 or cyclinB1 individually using two different siRNA oligonucleotides, with cyclinA2 siRNA having a stronger reductive effect (Fig. 5H; Figs S5F and S6), whereas cyclinA2+B1 siRNA together had an additive effect (Fig. 5I; Figs S5 and S6). These data show that cyclin–CDK1 complexes play an important role in facilitating invasive migration in both ovarian and bladder cancer cells, and that changes in expression of CDK1 and partner cyclins might contribute to the acquirement of an invasive phenotype in BC cells.

### CDK1 control of migration and invasion is independent of the cell cycle

**Fig. 6.**
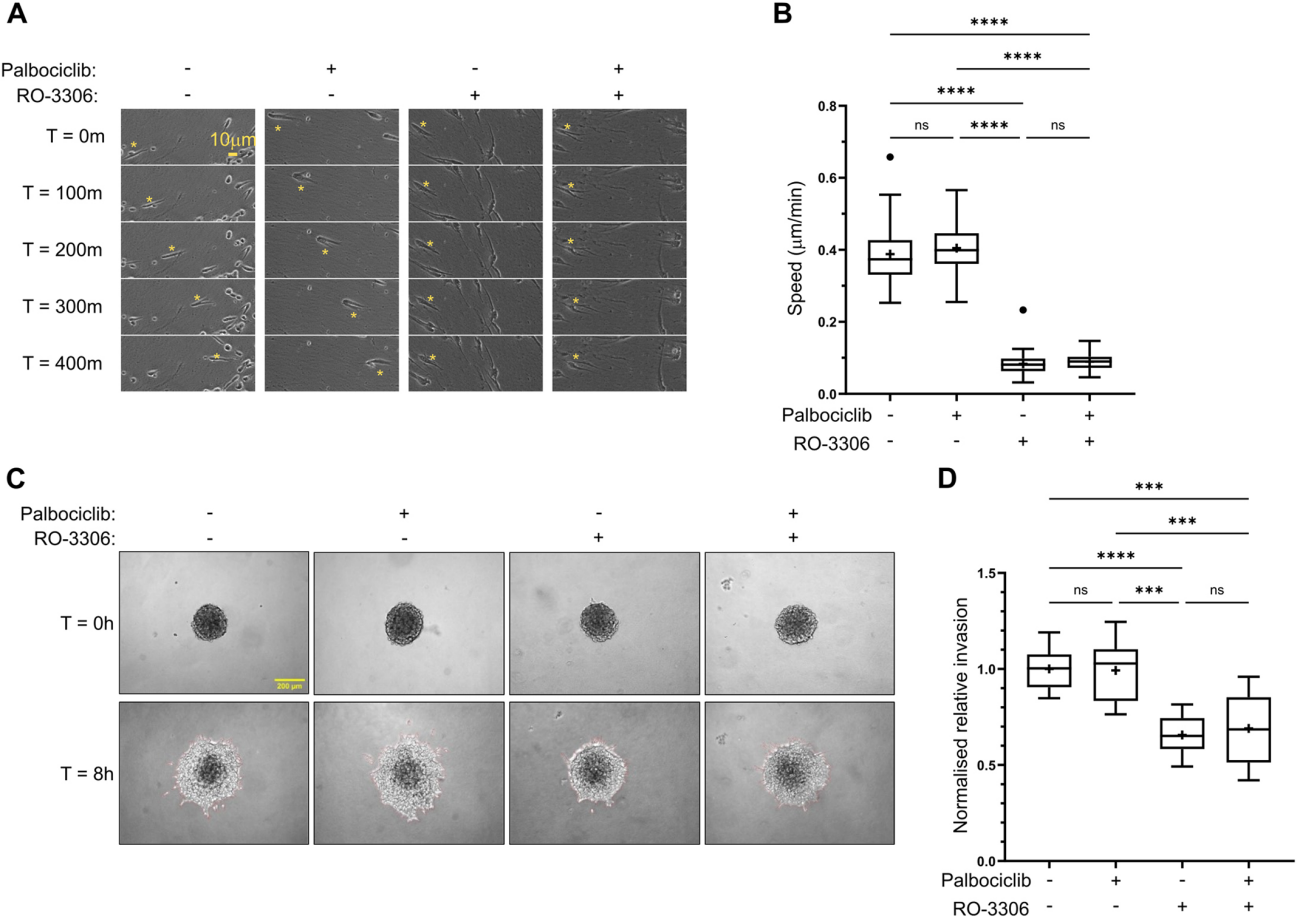
**Inhibition of CDK4/6 with palbociclib does not perturb cell migration and invasion in 3D matrices.** (A) A2780 cells seeded in CDM pre-treated (24 h prior to imaging) with CDK4/6 inhibitor palbociclib for arrest in G1 and/or CDK1 inhibitor RO-3306 (30 min prior to imaging) over 400 min time-lapse. The yellow asterisk denotes the position of the same cell at each time point. (B) Average migration speed of A2780 cells in CDM treated with different CDK1 and CDK4/6 inhibitor combinations across 16 h time-lapse. *n*=75 cells per condition analysed across three repeats. (C) T24 spheroid invasion assay showing T24 spheroids seeded in 2.5 mg/ml collagen gels treated with palbociclib and/or RO-3306 or DMSO at 0 h and 8 h timepoints. (D) Quantification of invasion of palbociclib- and/or RO-3306-treated spheroids relative to DMSO-treated controls. 9 spheroids per condition across three repeats was used for analysis. ****P<0.0001; ***P<0.001; ns, not significant (*P*>0.05) (ordinary one-way ANOVA with Tukey's multiple comparisons). In box plots, the box represents the 25–75th percentiles, and the median is indicated by the line and mean by the +. The whiskers show the furthest data points that are not considered outliers (1.5 times the interquartile range away from the box).

**Fig. 7.**
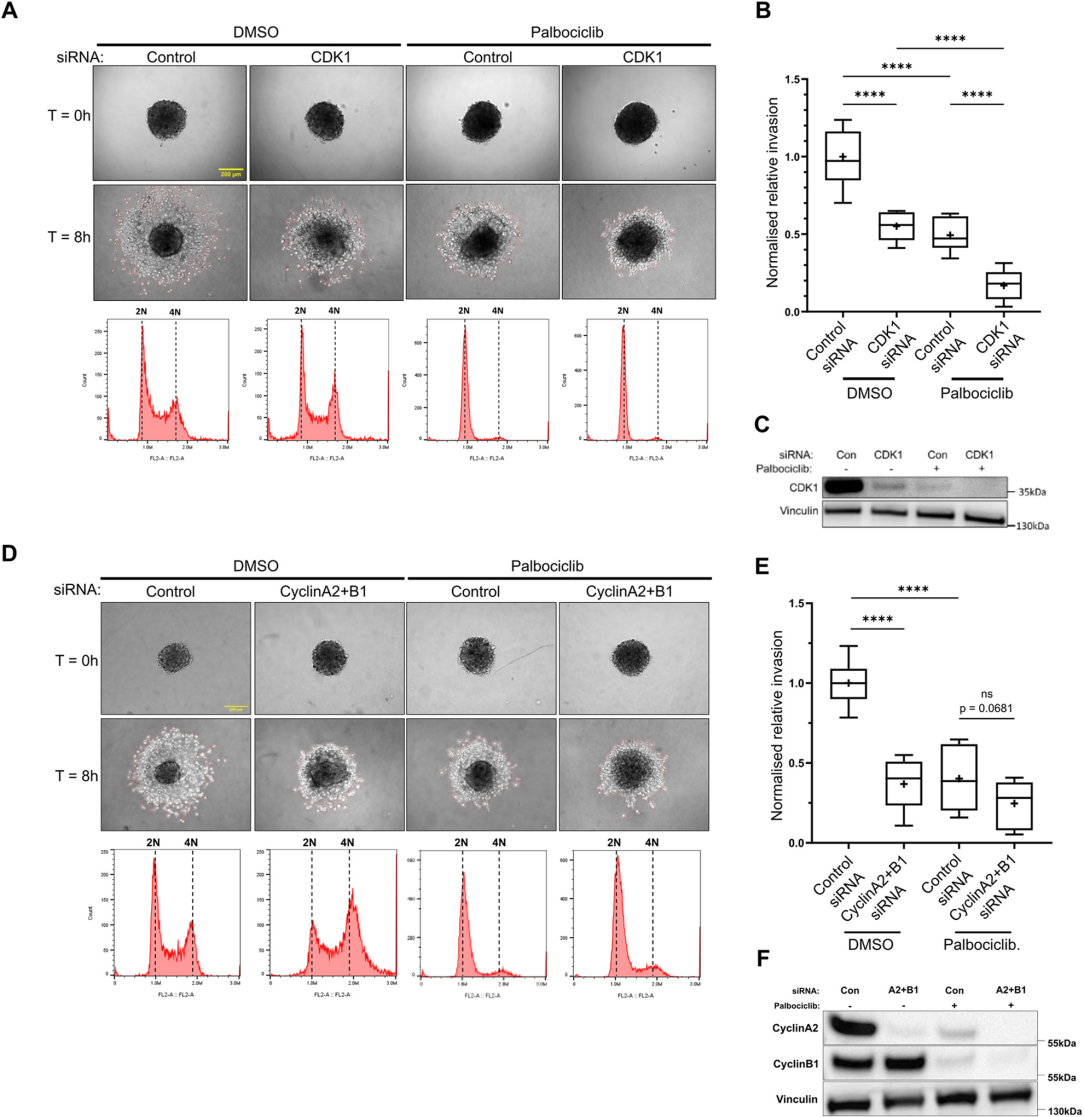
**CDK1, cyclinA2 and cyclinB1 regulate invasion in G0/G1 arrested cells.** (A) T24 cells induced into G1 arrest by pre-treating with palbociclib or DMSO (for controls) for 24 h followed by control siRNA treatment or CDK1 knockdown, while maintaining cells in DMSO or palbociclib (to maintain G1 arrest) for a total incubation time of 48 h. Spheroids are generated from knockdown cells and incubated for 18–24 h while maintaining G1 arrest using palbociclib or DMSO for controls. Representative images show generated T24 spheroids seeded into 2.5 mg/ml collagen gels pre-treated with DMSO or palbociclib (to maintain G1 arrest over duration of experiment) at 0 h and 8 h timepoints. Histograms shows cell cycle profile of propidium iodide-stained cells used to generate spheroids for each condition. 2N and 4N highlight diploid and tetraploid cell populations, respectively. (B) Quantification of invasion of CDK1 knockdown and palbociclib-treated spheroids relative to control siRNA DMSO controls. 9 spheroids per condition across three repeats was used for analysis. (C) Western blot showing protein levels of CDK1 in control siRNA and CDK1 siRNA T24 cells pre-treated with DMSO or palbociclib that were used for spheroid generation (quantified in Fig. S7C,D). (D) T24 cells induced into G1 arrest by pre-treating with palbociclib or DMSO (for controls) for 24 h followed by control siRNA treatment or cyclinA2+B1 knockdown, while maintaining cells in DMSO or palbociclib (to maintain G1 arrest) for a total incubation time of 48 h. Spheroids are generated from knockdown cells and incubated for 18–24 h while maintaining G1 arrest using palbociclib or DMSO for controls. Representative images show generated T24 spheroids seeded into 2.5 mg/ml collagen gels pre-treated with DMSO or palbociclib (to maintain G1 arrest over duration of experiment) at 0 h and 8 h timepoints. Histograms shows cell cycle profile of propidium iodide-stained cells used to generate spheroids for each condition. 2N and 4N highlight diploid and tetraploid cell populations, respectively. (E) Quantification of invasion of cyclin A2+B1 knockdown and palbociclib treated spheroids relative to control siRNA DMSO controls. 9 spheroids per condition across three repeats was used for analysis. (F) Western blot showing protein levels of CDK1 in control siRNA and CDK1 siRNA T24 cells pre-treated with DMSO or palbociclib that were used for spheroid generation (quantified in Fig. S7E,F). ****P<0.0001; ***P<0.001; ns, not significant (*P*>0.05) (ordinary one-way ANOVA with Tukey's multiple comparisons). In box plots, the box represents the 25–75th percentiles, and the median is indicated by the line and mean by the +. The whiskers show the furthest data points that are not considered outliers (1.5 times the interquartile range away from the box).

Having demonstrated that cyclin–CDK1 complexes are able to influence invasive cell migration using acute treatment with CDK1 inhibitors and longer-term depletion of CDK1 and cyclins, we next wished to determine whether the effects we were observing were due to a direct effect of CDK1 activity or an indirect effect of modulating the cell cycle. Treatment of cells with the CDK4 and CDK6 (collectively CDK4/6) inhibitor palbociclib results in a G0/G1 arrest that can be maintained over several days (Trotter and Hagan, 2020). Subsequently, this allows for knockdown of proteins to be undertaken in arrested cells, minimising any indirect effects of depleting CDK1, cyclinA2 and cyclinB1 might have on dividing cells. Short-term treatment of A2780 cells in CDMs or T24 cells in spheroid invasion assays with palbociclib (16 and 8 h, respectively) had no effect on cell motility or invasion (Fig. 6A–D; Movie 3), demonstrating that CDK4/6 does not play a direct role in regulating cell migration in these contexts. Despite short-term treatment with palbociclib altering the cell-cycle, complete arrest of cells in G1 did not occur (Fig. S7A); therefore, we treated cells for 24 h prior to undertaking CDK1 or double cyclin A2+B1 knockdown in the presence of palbociclib. This longer-term treatment (48 h) resulted in a complete G0/G1 arrest (Fig. S7A) and, in this context, knockdown of CDK1 or cyclin A2+B1 led to a reduction in T24 cell invasion, demonstrating that CDK1 and partner cyclins function in G0/G1-arrested cells to regulate motility (Fig. 7A–F). Interestingly, treatment with palbociclib for 24 h or above resulted in a reduction in cell invasion that is associated with a loss of CDK1, cyclinA2 and cyclinB1 (Fig. 7C,F; quantified in Fig. S7C–F and Fig. S7B). Taken together, these data suggest that the severe effect of CDK1–cyclin perturbation on cell motility does not extend to other cell cycle regulators, such as CDK4/6, and that CDK1, cyclinA2 and cyclinB1 have previously undiscovered non-canonical roles in migration and invasion, which are independent of cell cycle phase. Furthermore, these data demonstrate that palbociclib-induced G0/G1 arrest leads to a loss of cyclin–CDK1 complexes that potentially impacts upon the ability of cells to migrate in a 3D environment.

## DISCUSSION

In summary, our major findings identify differential roles for cyclin–CDK1 complexes in regulating cell motility and invasion in 3D matrices in normal and cancer cells. CyclinB1–CDK1 plays a specific role in regulating Ect2 localisation and activation of RhoA at the rear of migrating cells, thereby facilitating the contractile feedback loop that drives rear cell movement during motility (Hetmanski et al., 2019). In contrast, cyclinA2–CDK1 plays a broader role in regulating cell motility via its localisation at both the protrusive front and the rear of migrating cells. Depletion of cyclinB1 or cyclinA2 results in differing cell morphologies, with cyclinB1 depletion resulting in long, narrow cells indicative of a defect in rear retraction (Nguyen et al., 2018) whereas cyclinA2 depletion leads to the adoption of a large, rounded cell morphology. Knockdown of both leads to a combination of phenotypes that results in a complete abrogation of cell motility in addition to proliferation, alongside dramatic changes in cell size. These data define a role for cyclin–CDK1 complexes in regulating cell migration in addition to cell cycle progression. Furthermore, we observed increased expression of cyclinA2, cyclinB1 and CDK1 in invasive BC cells, in comparison to what was seen in non-invasive cells, suggesting that modulation of cyclin–CDK1 complexes might play a significant role in facilitating invasive cell migration in cancer.

A role for cyclinA2 and CDK1 in cell migration has been described in a range of cell types, including breast and hepatocellular cancer cells and Schwann cells (Chang et al., 2012; Fu et al., 2021; Lu et al., 2022) in addition to the cell types used in this study, demonstrating a conserved role for cyclinA2–CDK1 complexes in motility. In contrast, the role of cyclinB1–CDK1 in motility has not been previously described. We postulate that this is because cyclinB1–CDK1 plays a specific role in regulating the membrane tension-caveolae-RhoA contractility feedback loop at the rounded rear of cells that is only consistently observed in cells migrating within 3D matrices or in rigidity gradients (Hetmanski et al., 2019). The previous studies investigating the role of cyclins and CDK1 in migration have exclusively used 2D models of motility where the influence of cyclinA2–CDK1 on focal adhesions and the actin cytoskeleton dominates and the role of cyclinB1 might be less pronounced. This highlights the importance of considering how cell migration and invasion is regulated in 3D models. This same consideration applies to the localisation of cyclinA2, cyclinB1 and CDK1 observed in this study. CyclinA2 and CyclinB1 are differentially localised in cells cultured in 2D, with cyclinA2 being primarily nuclear and cyclinB1 being primarily cytoplasmic (Maridor et al., 1993; Pines and Hunter, 1991, 1994). In cell-derived matrices, this differential localisation is retained; however, cyclinA2 is observed more distinctly in the cytoplasm. Both cyclinA2 and cyclinB1 are trafficked in and out of the nucleus, with the differential localisation resulting from changes in import and export rates (Bendris et al., 2011; Hagting et al., 1999; Jackman et al., 2002; Yang et al., 1998) and it would be interesting to see whether these rates of nuclear translocation are altered in a substrate rigidity-dependent manner as for transcription factors, such as YAP1 (Dupont et al., 2011) or SRF and MAL (Connelly et al., 2010).

We have demonstrated here that depletion of cyclinB1 or CDK1 perturbs localisation of the RhoA GEF Ect2, and subsequently RhoA activity, at the rear of migrating cells and it will be interesting to determine whether direct phosphorylation of Ect2 by cyclinB1–CDK1 regulates Ect2 localisation and activity in motile cells. Ect2 is phosphorylated by cyclinB1–CDK1 during mitosis where it functions to activate RhoA to drive mitotic cell rounding and cytokinesis, as well as facilitating formation of the actin cortex in mitotic cells (Hara et al., 2006; Matthews et al., 2012; Niiya et al., 2006). We hypothesise that this role for cyclinB1–CDK1-dependent phosphorylation of Ect2 in regulating cortical actin and RhoA activity is conserved between mitotic and motile cells. Several other cytoskeletal regulators and focal adhesion proteins are phosphorylated during mitosis (Chen et al., 2022; Jones et al., 2019), suggesting that the conserved role of CDK1 in regulating the cytoskeleton is central to its function during mitosis, interphase and migration. Our data presented here and in our previous publications suggest distinct roles for cyclinB1– and cyclinA2–CDK1 complexes in cytoskeletal regulation, with the regulation of focal adhesions and phosphorylation of talin1 being primarily mediated by cyclinA2–CDK1 (Gough et al., 2021; Jones et al., 2018) and regulation of RhoA being driven by cyclinB1–CDK1. Identifying the cyclin–CDK1 complex specific mechanisms that regulate the cytoskeleton during cell cycle progression and migration within 3D environments, alongside determining any role for additional cell cycle regulators, therefore represents a significant avenue of future investigation. In particular, cyclinA2 can also associate with CDK2 and, although our data does not suggest a role for CDK2 in regulating ECM adhesion (Jones et al., 2018), it remains possible that CDK2 and other CDKs might play a role in regulating the cytoskeleton and motility in specific cell types and contexts.

In this study, we demonstrate a role for cyclin–CDK1 complexes in regulating cell migration in normal RPE1 cells in addition to invasive migration in ovarian and BC cells, suggesting that modulation of cyclinA2, cyclinB1 and CDK1 expression or cyclin–CDK1 complex activity might contribute to the acquisition of invasive capabilities in cancer cells. Here, we show that CDK1, cyclinA2 and cyclinB1 are expressed at increased levels in invasive BC cells in comparison to non-invasive cells, therefore identifying the regulatory mechanisms that control CDK1 and cyclin expression in cancer is paramount. Overexpression of CDK1 is observed in several tumour types (Wang et al., 2023), yet the control of CDK1 expression levels is poorly understood. We have shown here that prolonged inhibition of CDK4/6 with palbociclib and arrest in G0/G1 leads to a reduction in CDK1, cyclinB1 and cyclinA2. Despite this reduction, knockdown or inhibition of residual CDK1 perturbs migration and invasion in G1 arrested cells, suggesting that low levels of localised cyclin–CDK1 complexes are required to regulate motility and that cyclin–CDK1 complexes function to regulate motility independently of their role in cell cycle progression. Developing tools to directly measure localised cyclinA2–CDK1 activity and demonstrating that these low-levels of cyclin-CDK1 complexes are active and localised to the rear and front of G0/G1 cells migrating in 3D remains a priority. A reduction in cyclinA2 and B1 in palbociclib-treated cells is consistent with their well-studied regulation during cell cycle progression where levels are low in G1. Expression of cyclinA2 begins to increase during S-phase (Erlandsson et al., 2000; Pines and Hunter, 1990) and cyclinB1 increases during G2 (Minshull et al., 1990), before reaching maximal expression during mitosis and being subsequently degraded to facilitate mitotic exit (Clute and Pines, 1999; Geley et al., 2001). CDK1 levels, however, are widely considered to be consistent through the cell cycle and these data suggest that CDK4/6 might play a role in regulating CDK1 expression. In this regard, it is worth noting that a recent study using palbociclib to synchronise cells and study changes in protein expression during the cell cycle also observed a decrease in CDK1 following palbociclib treatment (Rega et al., 2025). These observations also raise the possibility that the use of CDK1 and CDK4/6 inhibitors to treat invasive cancer types such as ovarian and bladder cancer might be a viable therapeutic option as they will simultaneously block both cell proliferation and invasive cell migration. Determining how cyclin–CDK1 complexes are regulated and function in cancer models, such as organoids derived from individuals with diseases and mouse models, and how therapeutically targeting CDK1 influences cancer progression and metastasis in these models represents an important area of future investigation.

## MATERIALS AND METHODS

### Cell culture and transient transfection

A2780 human ovarian cancer cells (female) and RT4, J82 and T24 human BC cells (gifts from Prof. Ananya Choudhury, University of Manchester and The Christie NHS Foundation Trust, UK) were maintained in RPMI-1640 medium (Sigma-Aldrich) containing 0.3 g/l L-glutamine supplemented with 10% (v/v) fetal calf serum, and 1% (v/v) antibiotic-antimycotic (cat. no. A5955; Sigma-Aldrich); telomerase-immortalised fibroblasts (TIF) cells (gift from Prof. Jim Norman, CRUK Scotland Institute, UK; used to produce CDMs) were maintained in Dulbecco's modified Eagle's medium (DMEM, Sigma-Aldrich) containing 0.584 g/l L-glutamine and supplemented with 10% (v/v) fetal calf serum, and 1% (v/v) antibiotic-antimycotic (Sigma-Aldrich); human retinal pigment epithelial cells (RPE) cells (gift from Dr Sarah Woolner, University of Manchester, UK) were maintained in DMEM/Nutrient Mixture F-12 (DMEM/F12, Gibco) containing 0.584 g/l L-glutamine and supplemented with 10% (v/v) fetal calf serum and 1% (v/v) 100× non-essential amino acid solution (Sigma-Aldrich). Primary human urothelial cells (Sciencell) were maintained in Urothelial Cell Medium (Sciencell SC-4321) supplemented with urothelial cell growth supplement (Sciencell SC-4352) and 1% (v/v) penicillin-streptomycin. All cell lines were incubated at 37°C in a humidified 5% (v/v) CO_2_ atmosphere. siRNAs in A2780s and RPEs, and fluorescent constructs in A2780s, RPEs and T24s were transiently transfected by electroporation using a nucleofector (Amaxa, Lonza) using solution T, program A-23, 3 μg DNA/5 μl 20 mM siRNA as per the manufacturer's instructions. T24 cells were transfected with siRNAs using a Neon transfection system (Thermo Fisher Scientific) using Neon™ Resuspension Buffer R, 100 µl Neon™ Tips, pulse voltage 1400 V, pulse width 20 ms and pulse number 2, according to manufacturer's instructions. Experiments were performed ∼72 h after nucleofection for *CCNB1* and *CCNA2* and ∼48 h after nucleofection for CDK1 for all cell types, and ∼48 h after neon transfection for T24 cells.

### Reagents

Monoclonal antibodies used were: mouse anti-cyclinA2 [clone BF683, 1:1000 for western blotting (WB); 4656; Cell Signaling Technology], mouse anti-CDK1 (clone POH1, 1:1000 for WB; 9116; Cell Signaling Technology), rabbit anti-cyclinB1 [clone D5C10, 1:1000 for WB, 1:100 for immunofluorescence (IF); 12231; Cell Signalling Technology]; rabbit anti-cyclinD1 (clone E3P5S, 1:1000 for WB; Cell Signalling Technology); rabbit anti-p27/KIP1 (clone D69C12, 1:1000 for WB; Cell Signalling Technology) mouse anti-tubulin (clone DM1A, 1:10,000 for WB; ab7291; Abcam); mouse anti-vinculin (clone hVin-1, 1:2000 for WB; V9264; Sigma-Aldrich). Secondary Alexa Fluor 680-conjugated (1:10,000; A10043; Thermo Fisher Scientific), DyLight 800-conjugated (1:10,000; 5257; Cell Signaling Technology) and HRP-conjugated (1:10,000; SA1-100 and SA1-200; Thermo Fisher Scientific) antibodies were used for immunoblotting. Anti-mouse and anti-rabbit IgG Alexa Fluor 488-, 594- and 647-conjugated secondary antibodies (1:1000) and Alexa Fluor-tagged phalloidin were used for immunofluorescence (Thermo Fisher Scientific). Palbociclib and RO-3306 were purchased from Sigma-Aldrich, Flipper-TR was purchased from Spirochrome. The following plasmids used in this study were obtained as gifts: the FRET biosensor Raichu-1237X RhoA (Yoshizaki et al., 2003) was kindly provided by Prof. Michiyuki Matsuda (Kyoto University, Japan); the mCherry–Caveolin-1 construct (Hayer et al., 2010) was kindly provided by Dr Mark Bass (University of Sheffield, UK); Emerald-Lifeact was kindly provided by Prof. Christoph Ballestrem (University of Manchester, UK); BFP–Ezrin constitutively active (CA) (Gautreau et al., 2000) was kindly provided by Prof. Ewa Paluch (University of Cambridge, UK); GFP–Cavin-1 was kindly provided by Dr Jacky Goetz (Centre de Recherche en Biomédecine de Strasbourg, France); Lifeact-7-iRFP670 was Addgene plasmid #103032 (deposited by Ghassan Mouneimne); pLenti Lifeact-mTagBFP2 PuroR was Addgene plasmid #101893 (deposited by Ghassan Mouneimne). siRNAs used were: for *CDK1*, SMARTpool reagent L-003224-00- 0005, Horizon Discovery; for *CCNB1*, SMARTpool reagent L-003206-00-0005, Horizon Discovery or validated silencer select oligonucleotide s2517, Thermo Fisher Scientific; for *CCNA2*, validated silencer select oligonucleotides s2514 and s2513, Thermo Fisher Scientific; and for *CCNB2*, validated silencer select oligonucleotide s2517, Thermo Fisher Scientific.

### CDM generation

CDMs were generated using the method developed by the Yamada laboratory (Caswell et al., 2007; Cukierman et al., 2001). Six- or 12-well plastic (Corning) or 35 mm glass bottom (Mattek) plates were prepared by coating with 0.2% gelatin (v/v, Sigma-Aldrich) for 1 h, crosslinking with 1% glutaraldehyde (v/v, Sigma-Aldrich) for 30 min and quenching with 1 M glycine (Thermo Fisher Scientific) for 20 min before TIFs were confluently seeded. DMEM supplemented with 0.25% ascorbic acid (v/v, Sigma-Aldrich) was changed every 48 h for 8 days. Cells were denuded with extraction buffer [20 mM ammonium hydroxide (NH_4_OH) and 0.5% (v/v) Triton X-100] to leave only matrix before treatment with 10 µg/ml DNase 1 (Roche) to cleave phosphodiester linkages in the DNA backbone. CDMs were stored at 4°C with 1% (v/v) antibiotic-antimycotic (Sigma) and used within 3 months of generation.

### CDM migration

A2780 or RPE cells were seeded at sparse (∼50,000 cells/well) confluency in six-well CDMs and allowed to spread for ∼4 h. Images were acquired on an Eclipse Ti inverted microscope (Nikon) using a 20×/0.45 SPlan Fluar objective, the Nikon filter sets for Brightfield and a pE-300 LED (CoolLED) fluorescence light source with imaging software NIS Elements AR.46.00.0. Point visiting was used to allow multiple positions to be imaged within the same time-course and cells were maintained at 37°C and 5% CO_2_. The images were collected using a Retiga R6 (Q-Imaging) camera. Five randomly chosen positions per cell were captured every 10 min over 16 h (or 60 h for A2780 cyclinA2+B1 siRNA experiment) and five randomly chosen cells per position (meaning 25 cells tracked per condition per experiment) were individually manually tracked over the entire 16 h or first 16 h of the time-lapse using the ImageJ plugin MTrackJ every 3 frames (i.e. using 30 min timepoint intervals). Representative images of individual cells/fields of view are shown where appropriate.

Cell division percentages were quantified during the same time-lapse acquisitions as those used for migration in CDM. The total number of cells in the first frame of each position were manually counted, then all the division events during the first 16 h of the time-lapse were manually identified, with the ‘% dividing cells’ corresponding to (the total number of division events in 16 h/total cells at frame 1)×100. For analysis of cell speed before and after mitosis, cells were tracked as normal for the 6 h prior to contraction and rounding up for mitosis, and then the daughter cell continuing in the direction of the cell pre-mitosis was tracked for 6 h following respreading after mitosis.

For speed and cell size comparison analysis, 15 enlarging cyclinA2 siRNA A2780 cells were tracked as normal over the first 25 h of time-lapse acquisitions (as cells tended to move out of frame beyond this timepoint) and 18 cyclinA2+B1 siRNA A2780 cells were tracked as normal of the full 60 h time-lapse acquisition. The same cells were also manually analysed for cell width (the widest point of the cell) every 5 h for cyclinA2 siRNA and for width×length (the widest and longest points of the cell) every 10 h for cyclinA2+B1 siRNA and the direct comparison of average speed and average width or width×length plotted on the same graph. For the scatter plots, single points corresponding to the average speed in a 5 h period were plotted against the average cell width (e.g. the average of the width at the start and end of that 5 h time increment) for cyclinA2 siRNA cells, and single points corresponding to the average speed in a 10 h period were plotted against the average cell width×length in the same time increment for cyclinA2+B1 siRNA cells, and then simple linear regression calculated in Graphpad. For time-lapse graphs, the speed with respect to time and the width or width×length with respect to time were fitted with a quadratic curve (using Second order polynomial Least squares fit for speed of cyclinA2 siRNA cells and speed and width×length of cyclinA2+B1 siRNA cells) or linear regression (for width of cyclinA2 siRNA cells) in Graphpad to aid with visualisation. Researchers doing manual tracking were aware of the conditions as the phenotypes were severe and thus identifiable.

### Live-cell fluorescence imaging

All live-cell fluorescence images were acquired using a CSU-X1 spinning disc confocal (Yokagowa) on a Zeiss Axio-Observer Z1 microscope with a 63×/1.40 Plan-Apochromat objective for CDMs and a 40×/1.40 Plan-Apochromat objective for collagen gels. An Evolve EMCCD camera (Photometrics) and motorised *xyz* stage (ASI) was used. The 405, 488, 561 and 647 nm lasers were controlled using an AOTF through the laserstack [Intelligent Imaging Innovations (3i)] allowing both rapid ‘shuttering’ of the laser and attenuation of the laser power. Images were captured using SlideBook 6.0 software (3i). Randomly chosen representative polarised Lifeact–Emerald+mCherry–Caveolin-1, or Lifeact-iRFP670 expressing cells were captured with the appropriate excitation and emission spectrum, and exposure time following ∼4 h spreading time in CDM and ∼4 h spreading time in collagen gels in 1× Opti-Klear medium (Marker Gene Technologies) supplemented with 10% (v/v) FCS. Where indicated, cells were dyed with Hoechst 33258 for 10 min prior to imaging. Cells were imaged every 30 s for 5 min, and rear translocation in CDM was measured for all imaged cells in ImageJ across this 5 min period by comparing the position of a part of the rear of the cell at the start of the time-lapse with the exact same part of the rear at the end of the time-lapse. Rear-to-nucleus distance was determined in ImageJ by measuring the distance between the rearmost point of the nucleus to the rearmost point of the membrane at the first-time interval. For cell size and shape metrics in collagen gels, the ‘width’ and ‘length’ of the cells were manually determined at the widest and longest single positions in the cell, whereas aspect ratio is defined as length/width. For nucleus size, the Hoechst 405 channel was thresholded in ImageJ to create the nuclear mask, then the size of the mask determined by the measurement command. All cells in collagen gels were imaged live to determine polarity and movement direction and analysis was done on the 1st frame captured.

For quantification of Cav-1 intensity, a manual macro was written in ImageJ (available upon request). On the first frame of time-lapse movies, the 488 Emerald–Lifeact channel was used to threshold the cells, with background pixels assigned NaN and the fill holes command used to include the whole cells in the quantified region. The thresholded Emerald–Lifeact channel was then multiplied by the mCherry–Cav1 channel so that all the pixels within the cell had a Cav-1 intensity value and all the background pixels were set to NaN. Then the line profile from the rearmost point of the cell forward of this Cav-1 intensity was measured for a line wider than the total width of the cell, such that each value represented the average Cav-1 intensity across the entire width of the cell for that position relative to the rear. The rearmost 20 values (corresponding to ∼9 µm from the rearmost point of the cell forward) were then averaged to define the ‘rear’ and all the values from the 21st value forward were averaged as the ‘rest’, and the ‘rear/rest’ ratio plotted.

### Staining and fixed imaging

Cells were fixed in 4% paraformaldehyde (PFA) at room temperature following ∼4 h spreading in CDM after non-confluent (∼50,000 cells/plate) seeding. For osmotic shock, medium was replaced with 50% 1× RPMI, 50% distilled water 30 min prior to fixation, while medium was replaced at the same time with 100% 1× RPMI in the control isotonic condition. Membranes were permeabilised with 0.2% (v/v) Triton-X and blocked in 5% (w/v) heat-denatured bovine serum albumin (BSA) before being stained with appropriate antibodies as in reagents section. Cells were incubated with secondary antibodies rabbit Alexa Fluor 647-conjugated and mouse Alexa Fluor 594 conjugated for 1 h, (both Invitrogen), stained with Phalloidin Alexa Fluor 488-conjugated (Invitrogen) for 1 h, and Hoechst 33258 (Thermo Fisher Scientific) for 10 min.

Cells were imaged using a Leica TCS SP8 STED microscope with an HC PL APO 100×/1.40 oil objective using a HyD1 detector, and a notch filter was used for background reduction where possible. Images were captured using a white light laser (WLL) with excitation wavelengths 488, 561 and 633 nm and appropriate emission spectra for green, red and far red, respectively; a Diode 405 laser was used with appropriate emission spectra for blue. *Z*-stacks were captured covering the entire *z*-profile of the cell with intervals of 0.3 μm at a zoom of 1×. All subsequent analysis and quantification was performed on maximum intensity projections (MIPs) in ImageJ; all representative images shown throughout are MIPs [pseudocoloured using the ‘red hot’ look-up table (LUT) where appropriate to reveal differences in intensity].

For quantification of localised CDK1, cyclinA2 and cyclinB1 intensity, images were analysed with the interactive 3D surface plot v2.31 in ImageJ using a 128-grid size and smoothing factor of 3.0. Under these conditions, the peak intensities of the rear, front and nuclear cell regions were manually identified, and the ratio rear peak/non-rear peak or rear peak/nuclear peak was calculated, therefore providing inherent normalisation, avoiding staining and expression discrepancies. The rear peak/non-rear peak ratios between conditions were then directly compared.

### Collagen gel generation for single-cell imaging

Collagen gels were generated using an adapted approach to collagen gel generation as used in inverted invasion assays described previously (Hennigan et al., 1994; Hetmanski et al., 2016). 10× RPMI, water, NaHCO_3_, NaOH were mixed and added in equal volume to 3 mg/ml collagen (Gibco) and supplemented with 25 μg/ml fibronectin (F1141 Sigma), while being kept on ice to prevent polymerisation. Polymerised rat tail collagen (Gibco) was labelled with Alexa-488 NHS Ester (A2000, Thermo Fisher Scientific) before being solubilised and added at a 1:10 ratio with unlabelled collagen. ∼50,000 cells of interest per condition (A2780 or RPE cells previously transfected with siRNA as above) were centrifuged at 300 ***g*** for 4 min and seeded directly into the collagen gel mix (while still liquid, pre-polymerisation). 50 µl of the cells and collagen mixture was pipetted onto the glass area of a 35 mm Mattek dish (to cover the circular glass surface) and the collagen was allowed to polymerise for 30 min at 37°C. Dishes were then gently agitated to detach the gels from the glass area (to ensure cells in 3D collagen and not adhered to the glass surface below) and normal growth medium added. Cells were imaged 16 h post seeding. For Flipper-TR experiments, Flipper-TR (Spirochrome, SC020) was added at 1:250 following collagen addition (prior to cell seeding and polymerisation) and cells were imaged ∼4 h post seeding.

### RhoA FLIM imaging

Cells previously transfected with GFP–RFP Raichu RhoA were seeded into CDM 4 h prior to imaging and imaged at 37°C in 1× OptiKlear with 10% FCS. Cells were imaged on a Leica SP8 gSTED microscope using Leica FALCON hardware/software for fluorescent lifetime imaging (FLIM) of the GFP donor channel with 488 nm excitation and 498–550 emission with speed 50 Hz, line average 6 and format 512×512 pixels. Following imaging, images were subjected to 2×2 binning (to improve signal to noise). For quantification, regions at the cell rear and cell front were manually identified and drawn in the Leica FALCON software, and the mean average lifetimes measured by fitting a single exponential to these front and rear regions of interest (ROIs). For the lifetime representative images, the lifetime limits were set to 1–2 ns, the counts limits to 0–2000 and the images exported with the default Leica LUT.

### FlipperTR FLIM imaging

Cells were seeded directly into collagen gels containing 4 μM Flipper TR 4 h prior to imaging and imaged at 37°C in 1x OptiKlear (without FCS). Cells were imaged on a Leica SP8 gSTED microscope using Leica FALCON hardware and software for fluorescent lifetime imaging (FLIM) with 488 nm excitation and 575–625 emission with speed 50 Hz, line average 6 and format 512×512 pixels. Following imaging, images were subjected to 2×2 binning (to improve signal to noise) and a double exponential was fit to ensure a close fit (chi squared <1.5). For quantification, membrane regions at the cell rear and cell front were manually identified and drawn in the Leica FALCON software, and the mean average lifetimes measured by fitting a single exponential to these front/rear ROIs. For the lifetime representative images, the lifetime limits were set to 3–5 ns, the counts limits to 0–1000 and the images exported with the default Leica LUT.

### Inverted invasion assay

Inverted invasion assays were performed based on the protocol as described previously (Hennigan et al., 1994). ‘Stiff’ (final concentration 5 mg/ml; Corning) or ‘soft’ (final concentration 1.5 mg/ml, Gibco) Collagen I supplemented with 25 μg/ml fibronectin was allowed to polymerise in inserts (Transwell; Corning) for 1 h at 37°C upon addition mixing with 10× RPMI, water, NaHCO_3_ and NaOH to regulate pH. Transwell inserts were then inverted and ∼50,000 cells were seeded directly onto the bottom surface for 4 h at 37°C. Transwell inserts were then re-inverted, washed and placed in serum-free medium. Medium supplemented with 10% FCS and 30 ng/ml EGF (Miltenyi Biotec), was placed on top of the matrix to provide a chemotactic gradient for invasion. After 72 h, all cells were stained with Calcein-AM (C1430, Thermo Fisher Scientific) at ∼1 h prior to imaging and visualised by confocal microscopy with serial optical sections being captured at 15-μm intervals using an inverted confocal microscope (SP8 gSTED, Leica) using a 20× objective. Invasion was quantified using the area calculator plugin in ImageJ, where the invasive proportion was obtained by measuring the fluorescence intensity of cells invading >45 μm the 5th *z*-stack onwards and dividing this by the total fluorescence intensity in all *z*-stack images (15 planes in total).

### Immunoblotting

Cells were lysed in lysis buffer [200 nM NaCl; 75 mM Tris; 15 mM NaF; 1.5 mN Na_3_VO_4_; 7.5 mM EDTA; 7.5 mM EGTA; 1.5% (v/v) Triton X-100; and Igepal CA-630] supplemented with Halt™ protease (100×) and phosphatase (100×) inhibitor cocktail (Thermo Fisher Scientific). Lysates were clarified by centrifugation at 10,000 ***g*** for 20 min at 4°C.

Cell lysates were separated by SDS-PAGE (4–12% Bis-Tris gels; Thermo Fisher Scientific) under reducing conditions and transferred onto nitrocellulose membranes (Cytiva). Membranes were blocked for 60 min at room temperature (RT) using casein-blocking buffer (Sigma-Aldrich) and then probed overnight with primary antibodies diluted in TBST (110 mM Tris-HCl, pH 7.4, 150 mM NaCl, 0.05% Tween-20) at 4°C. Membranes were washed for 15 min by using TBST and then incubated with the appropriate secondary antibodies diluted in TBST for 90 min at RT. Membranes were washed for 15 min by using TBST and exposed to chemiluminescent substrate and bound antibodies were visualised using a Syngene G:BOX CHEMI XRQ (Syngene) for T24, RT4, HUC, J82 and UMUC3 lysates; or scanned using an infrared imaging system (Odyssey; LI-COR Biosciences) for A2780 and RPE lysates. Band intensities normalised to loading controls were analysed using ImageJ throughout.

### 3D spheroid invasion assay

Spheroids were generated using RT4 and T24 BC cells utilising the hanging-drop method. Cells were suspended in complete RPMI and methocel solution [12 mg/ml methylcellulose (Sigma-Aldrich M7027) diluted in complete RPMI] at a 4:1 ratio. Cell suspensions were added to the under-side of a Petri dish lid in 20 µl droplets at a density of 2000 cells per droplet and inverted onto the base of a Petri dish containing 5 ml PBS to prevent spheroids from drying out. Cells aggregated on the bottom of 20 µl droplets due to gravity and formed compact spheroids after 18–24 h. Collagen gels were generated using PureCol™ EZ Gel (Sigma-Aldrich) by diluting in neat RPMI to a concentration of 2.5 mg/ml and stored at 4°C. In a 96-well plate, 40 µl of 2.5 mg/ml collagen gel was added to each well and incubated at 37°C for 24 h alongside spheroids to allow for sufficient gelation to ensure spheroids would not sink and form a monolayer on the bottom of the well. Following spheroid generation, an additional 60 µl of 2.5 mg/ml collagen gel was added to initial 40 µl gel layers and spheroids in hanging droplets were removed from droplets in a volume of 5 µl using a p20 pipette and injected directly into 60 µl collagen gel layer. Spheroids in gels were first imaged at 20× magnification using a DMi8 inverted microscope (Leica) and then incubated for 90 min to allow sufficient gelation, following which 100 µl of complete RPMI was added to each gel. Spheroids were then incubated further at 37°C to a total incubation time of 8 h before being imaged again using the DMi8 inverted microscope. Area of invasion was measured using ImageJ and determined by measuring initial spheroid area at 0 h and subtracting value from total area following 8 h invasion into collagen gel.

For CDK drug treatments, collagen gels were additionally supplemented with either DMSO, 2 µM palbociclib or 10 µM RO-3306 (1:1000 dilution from stock solutions).

### Growth curve analysis

BC cells were seeded into wells of a 96-well plate at a density of 2.5×10^3^ and incubated at 37°C for up to 72 h. At indicated time-points (0, 24, 48 and 72 h), cells were pulsed with 4 µM Calcein-AM (Dojindo) and incubated at 37°C for 1 h to allow for sufficient esterase activity. The number of viable cells was determined by measuring fluorescence at an excitation wavelength of 485 nm and an emission wavelength of 520 nm using a FLUOstar Omega Microplate reader (BMG Labtech).

### Flow cytometry

For cell cycle analysis, cells were washed once with phosphate-buffered saline (PBS) and released from the substrate with trypsin. Cells were pelleted and washed in PBS before fixation in ice-cold ethanol and stored at −20°C for a minimum of 24 h. Fixed cells were pelleted (500 ***g*** for 5 min) and washed three times in PBS at RT before a final pellet (500 ***g*** for 5 min) was resuspended in 500 µl FxCycle™ PI/RNase Staining Solution (Thermo Fisher Scientific) and incubated at RT for a minimum of 30 min. Samples were ran on a Accuri™ C6 Flow Cytometer (BD Biosciences), with a total of 10^4^ cells counted for each sample. Data were analysed with FlowJo™ software (BD Biosciences).

### EDU proliferation assay

BC cells were cultured on coverslips at a density of 4.5×10^4^ cells and incubated at 37°C for 24 h. Following 24 h incubation, cells were pulsed with 10 µM EDU and incubated at 37°C for a further 2 h. The prepared cells were washed three times in PBS and fixed with 4% paraformaldehyde for 15 min at room temperature, followed by membrane permeabilisation in 0.2% Triton X-100/PBS for 10 min and blocking with 1% BSA supplemented with 0.1 M Glycine to quench fixative for 30 min. Cells were EdU-labelled using Click-it chemistry according to the manufacturer's instructions (Thermo Fisher Scientific) and counter stained with Hoechst 33342 (Thermo Fisher Scientific). Images were acquired using a Leica SP8 confocal microscope with a 20× objective and Leica LAS X Software (Leica). Image analysis was performed using ImageJ.

### GEPIA data analysis

Gene expression analysis was performed using GEPIA (Gene Expression Profiling Interactive Analysis), available at http://gepia.cancer-pku.cn/index.html. The Expression DIY tool was used to generate box plots that profile the expression levels of specific cell cycle genes across bladder tumour and normal bladder tissue samples from the TCGA and GTEx databases. Boxplots were generated automatically following selection of the following parameters: gene of interest, bladder cancer (tissue type), a Log_2_FC cutoff value of 1, a *P*-value cutoff value of 0.01, and log_2_(TPM + 1) was used for the *y*-axis. Boxplots show bladder tumour samples in red and normal bladder samples in grey with a star indicating statistical significance between each condition.

## Supplementary Material

10.1242/joces.263697_sup1 Supplementary information
