# Supplementary material for: Differential roles of cyclin–CDK1 complexes in cell migration and invasion
Source: J Cell Sci. 2025 Jul 14;138(13):jcs263697. doi: 10.1242/jcs.263697 (PMC12301658; doi:10.1242/jcs.263697)
Supplement: Supplementary information [file joces-138-263697-s1.pdf]

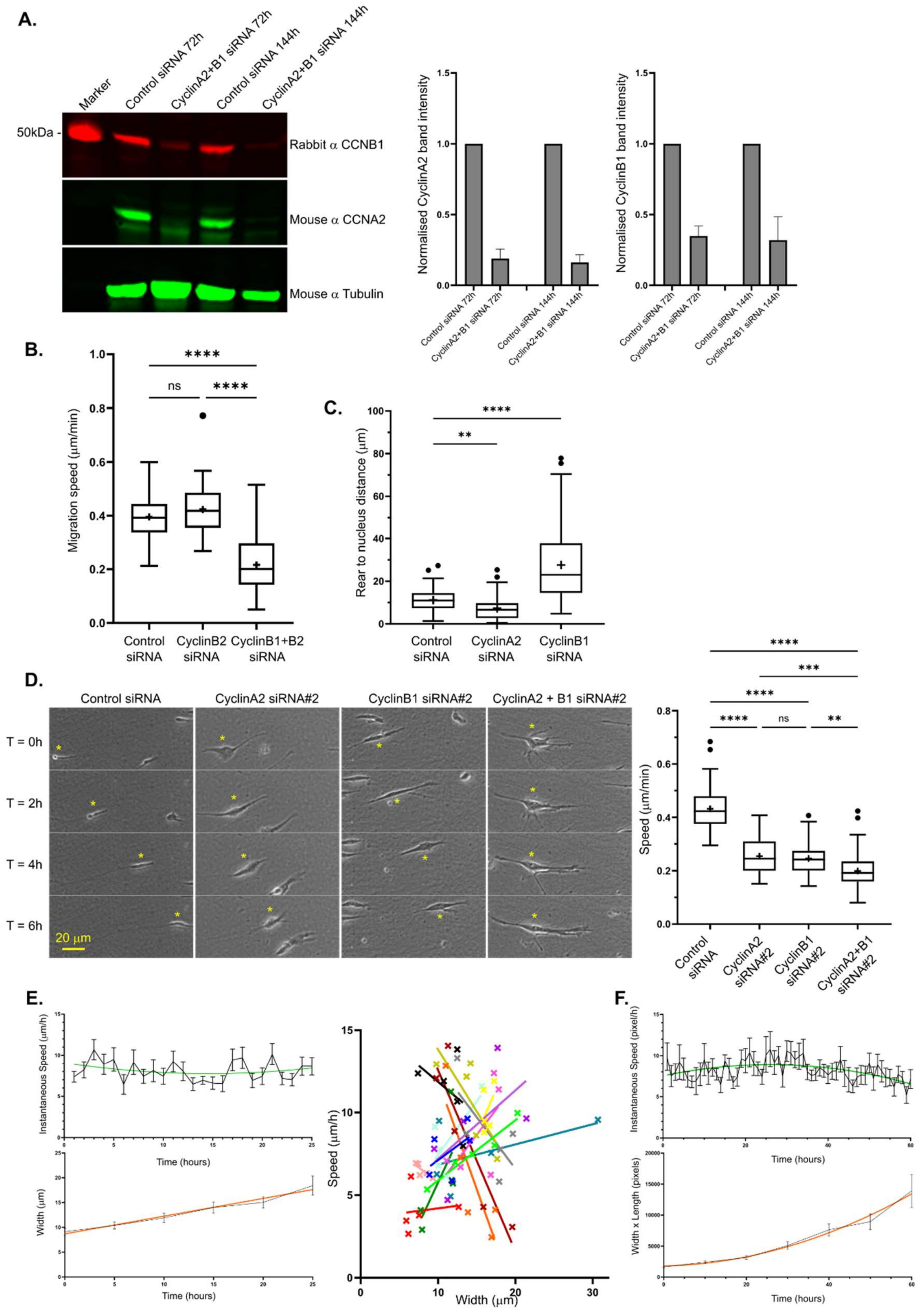

Fig. S1. Knockdown of cyclinA2 and cyclinB1 alters nuclear positioning in migrating cells, knockdown of cyclinB2 has no effect on cell migration, 2nd independent siRNA effect on cell migration and speed/size comparison in enlarging cells. A. (left) Western blot showing cyclinA2

(CCNA2), cyclinB1 (CCNB1) and Tubulin (for loading) protein levels for control siRNA and cyclinA2 +B1 siRNA A2780 samples corresponding to the start of the longterm timelapse imaging (72h) and at the end of the longterm timelapse imaging (144h); (right) quantification of cyclinA2 and cyclinB1 band intensity on Western blots normalised to control siRNA cells at the corresponding time point, SEM shown across 3 repeats **B.** Average distance between the nucleus and cell rear of control, cyclinA2 and cyclinB1 siRNA A2780 cells (as determined using the lifeact channel overexposed and the rearmost nucleus and rearmost membrane points in the first frame), n > 40 cells per condition across 3 repeats. **C.** Average migration speed of cyclinB2 and concomitant cyclinB1+B2 siRNA A2780 cells in CDM across 16 hour timelapse, n = 50 cells per condition analysed across 3 repeats**. D.** (left) Control, cyclinA2, cyclinB1 and cyclinA2 + B1 knockdown A2780 cells seeded in CDM migrating over 6 hours, yellow * denotes the position of the same cell at each time point; (right) Average migration speed of individual control, cyclinA2, cyclinB1 and cyclinA2 + B1 siRNA A2780 cells in CDM across 16 hour timelapse, n = 75 cells per condition analysed across 3 repeats, different siRNAs for cyclinA2 and cyclinB1 used than in Figure 1A-C. **E.** (left) Instantaneous speed each hour (top) and width (bottom) of enlarging cyclinA2 cells over 25 hour time period, with quadratic curve fit for speed (green line) and linear regression fit for width (orange line); (right) Speed plotted against width for cyclinA2 siRNA cells over 5 hour time increments as in Figure 1L with individual cells colour coded and individual linear regression fitted for each cell. **F**. Instantaneous speed each hour (top) and width x length (bottom) of enlarging cyclinA2 + B1 siRNA cells over **60** hour time period, with quadratic curves fitted for speed (green line) and for width x length (orange line); One way ANOVA compared to control used in B, C and D, **** denotes p < 0.0001, *** denotes p < 0.001, ** denotes p < 0.01, ns denotes p > 0.05 (not significant).

Journal of Cell Science • Supplementary information

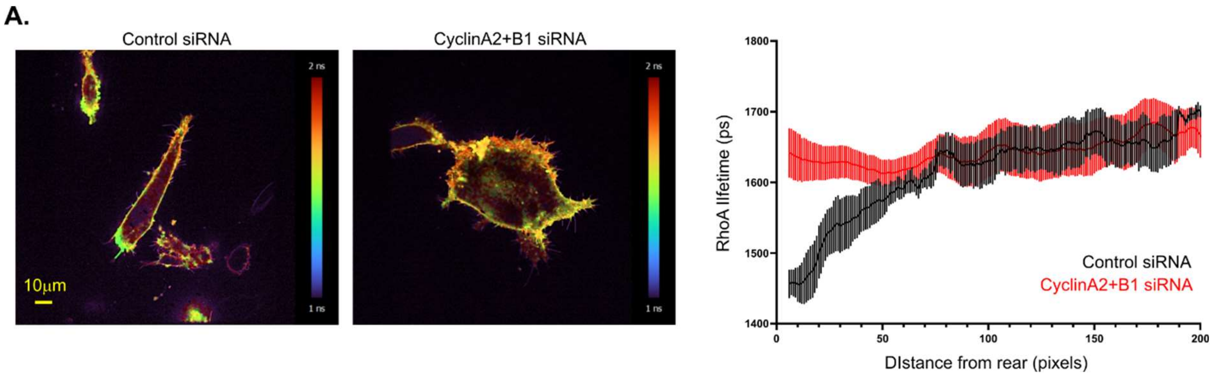

Fig. S2. Knockdown of cyclinA2a nd cyclinB1 perturbs activation of RhoA at the rear of migrating cells and cell migration speeds to not change either side of mitosis. A.C ontrol and cyclinA2 + cyclinB1 concomitant knockdown A2780 cells seeded in CDM transfected with GFP-RFP Raichu-RhoA probe, FLIM lifetime of donor GFP channel shown, blue denotes shorter lifetime (high activity), yellow/red denotes higher lifetime (low activity), colourbar shown. Average lifetime across the entire width of the membrane per pixel from the rear, (averaged to nearest 10 front-rear length pixels) for control (black line/bars) and cyclinA2 + cyclinB1 siRNA (red line/ bars) A2780s in CDM; standard error mean (SEM) shown, averaged across >12 cells for each condition.

Journal of Cell Science • Supplementary information

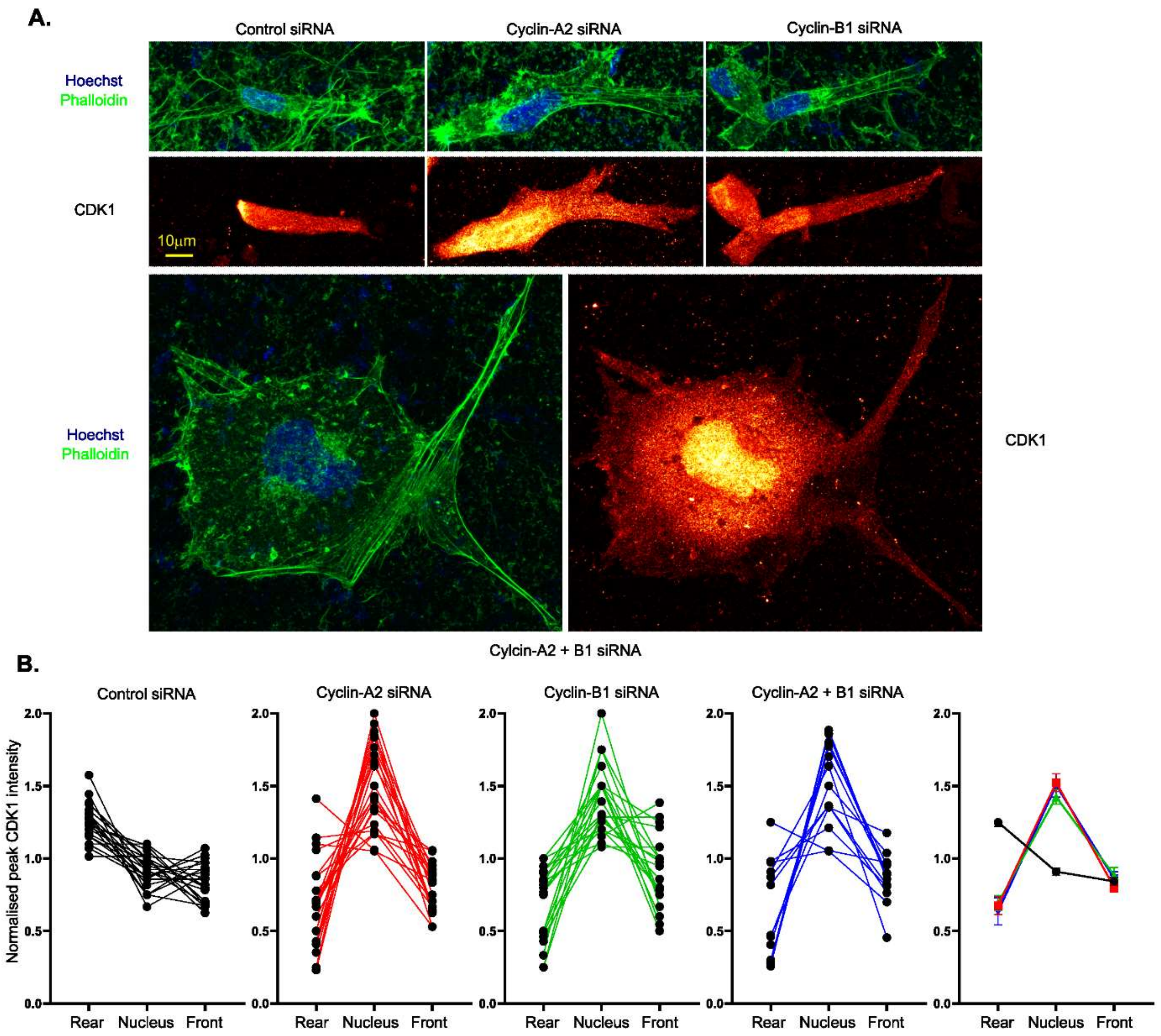

Fig. S3. Knockdown of cyclinA2 or cyclinB1 perturbs CDK1 rear localisation in migrating cells. A. Control, CyclinA2, CyclinB1, and cyclinA2 + cyclinB1 concomitant knockdown A2780 cells fixed 4 hours post seeding in CDM, stained with 488-phalloidin for actin (green, top/left images), Hoechst 33258 for nucleus (blue, top/left images) and CDK1 (Mouse αCDK1 primary, α Mouse Cy3 secondary, red hot look-up table applied, bottom/right images). **B.** Normalised CDK1 staining intensity in rear, nucleus and leading edge manually identified regions of A2780s in CDM; left 4 graphs for each condition as indicated where each connected line represents a single cell, right graph shows collated data from all cells for each condition with error bars representing SEM.

Journal of Cell Science • Supplementary information

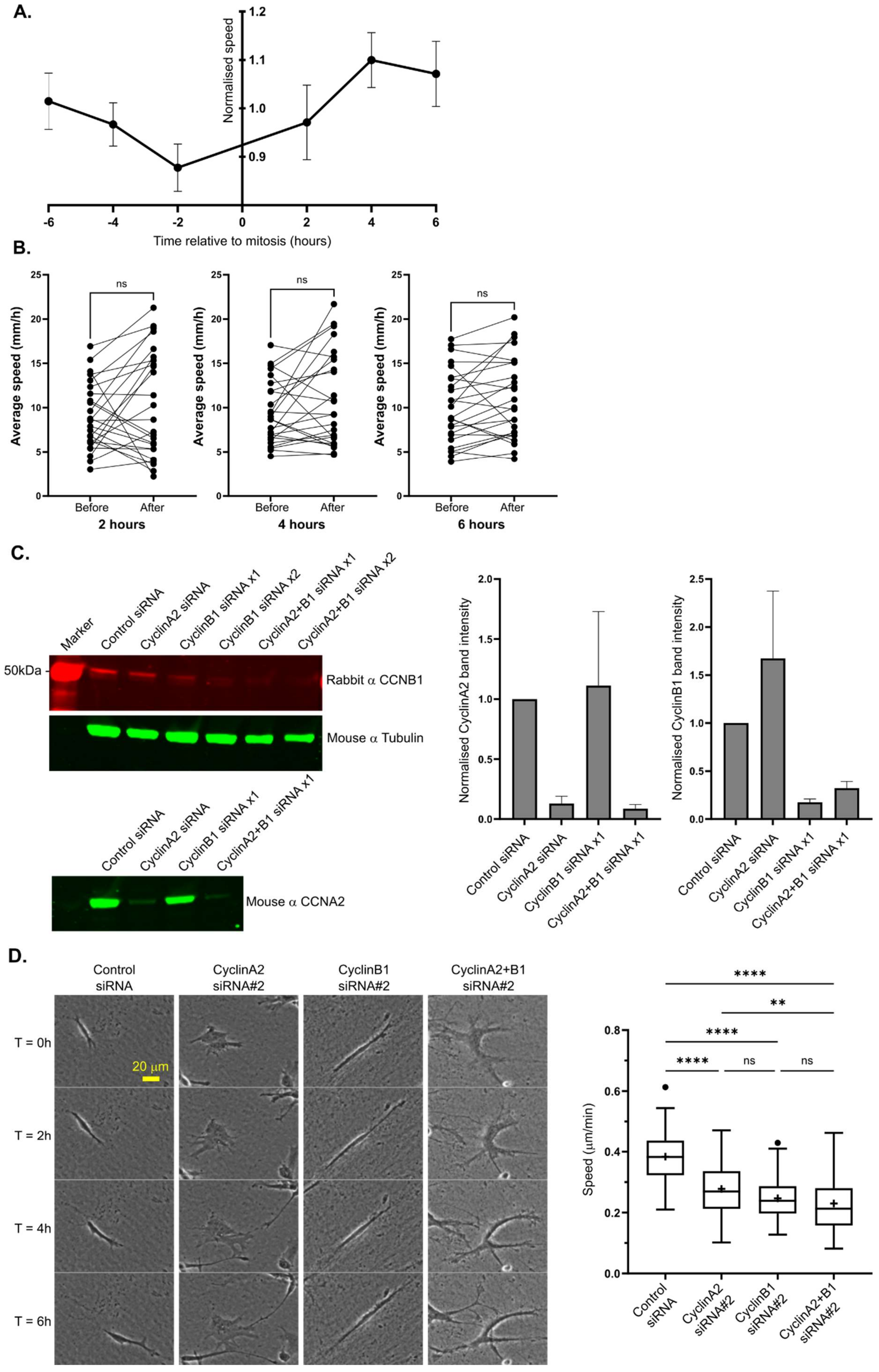

Journal of Cell Science • Supplementary information

Fig. S4. Representative Western blots showing cyclin knockdowns in RPE cells and second independent siRNA effect on migration. A. Average migration speed of control RPE cells in two hour intervals prior to mitosis and two hour intervals after respreading post mitosis, 18 cells analysed. **B.** Pairwise comparisons of cell migration speeds 2 hours (left), 4 hours (centre), and 6 hours (right) before and after mitosis, same 18 cells analysed as in B, same cells joined by black line. Paired student t tests used in C, ns denotes P > 0.05.**C. (**Top) Labelled western blot showing cyclinA2 (CCNA2), cyclinB1 (CCNB1) and Tubulin (for loading) protein levels for control, cyclinA2, cyclinB1 and cyclinA2+B1 siRNA RPE samples corresponding to the start of the longterm timelapse imaging (72h), x1 denotes single knockdown, x2 denotes knockdown performed on consecutive days 24 hours apart; (bottom) quantification of cyclinA2 and cyclinB1 band intensity on Western blots normalised to control siRNA cells, SEM shown across 3 repeats. **D.** (Left) Control, cyclinA2, cyclinB1 and cyclinA2 + B1 knockdown RPE cells seeded in CDM migrating over 6 hours, yellow * denotes the position of the same cell at each time point; (right) Average migration speed of individual control, cyclinA2, cyclinB1 and cyclinA2 + B1 siRNA RPE cells in CDM across 16 hour timelapse, n = 75 cells per condition analysed across 3 repeats, different siRNAs for cyclinA2 and cyclinB1 used than in Figure 4A-C.

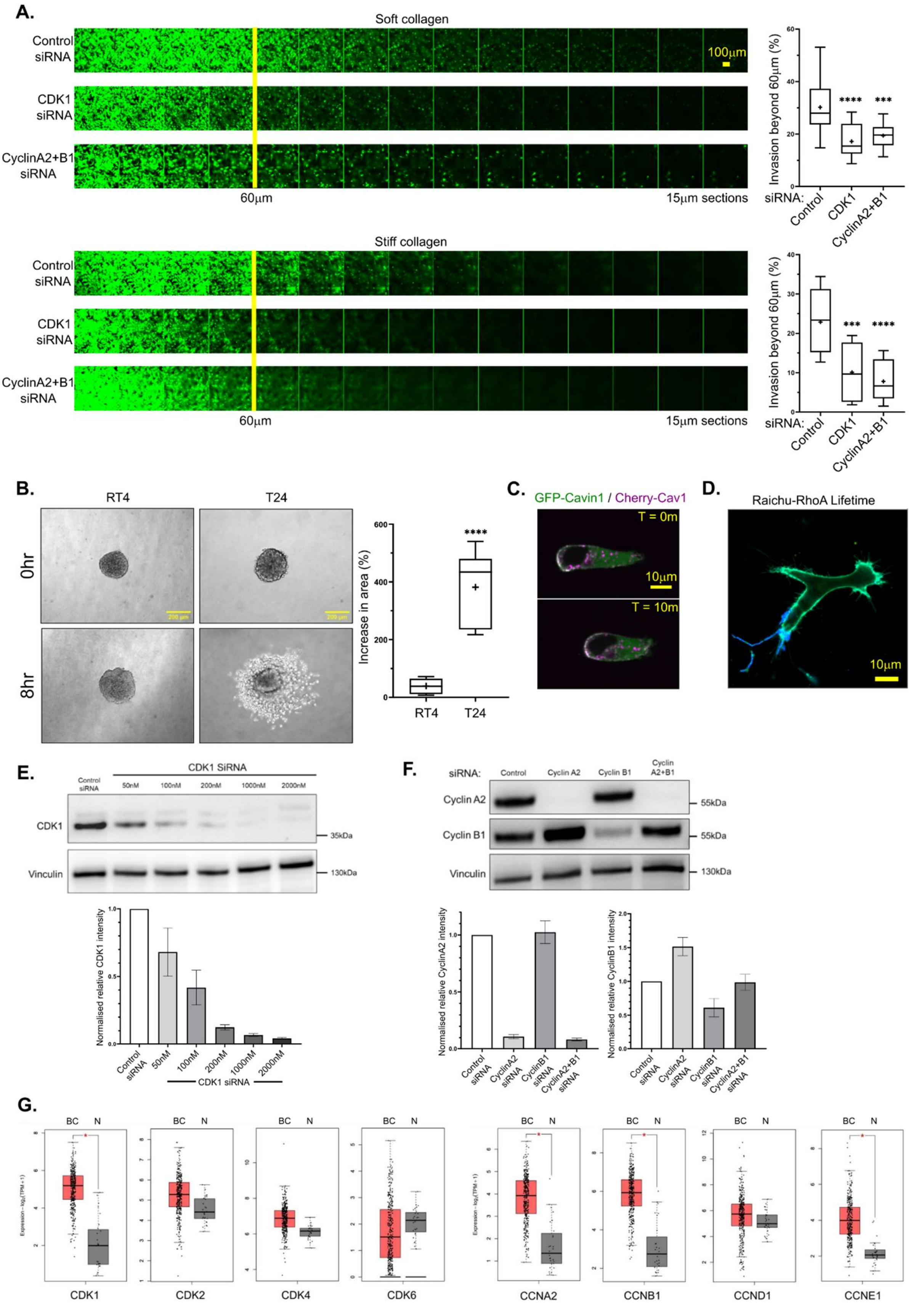

Figure S5: Knockdown of CDK1, cyclinA2 and cyclinB1 perturb ovarian and bladder cancer cell invasion in 3D collagen gels. A. Left: Representative montage images of Z planes from inverted invasion assay using A2780 cells stained with calcein-AM 1h prior to imaging, each image- taken 15mm apart whereby cells which are visible beyond the 4th slice (60mm) are taken as invasive for cells in 'soft' (final

Journal of Cell Science • Supplementary information

Fig. S5. Knockdown of CDK1, cyclinA2 and cyclinB1 perturb ovarian and bladder cancer cell invasion in 3D collagen gels. A. Left: Representative montage images of Z planes from inverted invasion assay using A2780 cells stained with calcein-AM 1h prior to imaging, each image- taken 15mm apart whereby cells which are visible beyond the 4th slice (60mm) are taken as invasive for cells in 'soft' (final concentration 1.5mg/ml, top) and 'stiff' (final concentration 5mg/ml, bottom) collagen; right: Quantification of % of cells which have invaded beyond 60mm, calculated as the total thresholded intensity in beyond the 4th slice / the total thresholded intensity of the whole stack, n=9 fields analysed per condition across 3 repeats. **B.** Spheroids generated from invasive T24 or non-invasive RT4 cells seeded into 2.5mg/ml collagen gels at 0h and 8h timepoints. Quantification (right) of percentage increase in area of T24 spheroids relative to RT4 spheroids following 8h incubation, n=9 spheroids analysed per condition across 3 repeats. **C.** Representative T24 cell transfected with GFP-Cavin1 (green) and cherry-caveolin-1 (magenta, white overlap indicated caveolae localisation) seeded in CDM at two time points 10 minutes apart. **D.** Representative T24 cell transfected with GFP-RFP Riachu-RhoA, lifetime of FLIM image of GFP donor channel shown where blue denotes shorter lifetime, higher RhoA activity. **E.** Western blot showing changes in CDK1 protein levels of T24 cells following treatment with varying concentrations of CDK1 siRNA used in spheroid invasion assay. Quantification (below) of CDK1 protein levels relative to control siRNA normalised to vinculin. Error bars show SEM. **F.** Western blot showing cyclinA2 and cyclinB1 protein levels in T24 cells following treatment with control, cyclinA2, cyclinB1 or a combination of cyclinA2+B1 siRNA. Quantification (below) of cyclinA2 (bottom/left) and cyclinB1 (bottom/right) protein levels relative to control siRNA normalised to vinculin. Error bars show SEM. **G.** Gene Expression Profiling Interactive Analysis (GEPIA; http://gepia2.cancer-pku.cn/) highlighting expression levels of selected CDK's and their respective cyclins in bladder cancer tissue (BC) compared to normal tissue (N). Red boxes correspond to bladder cancer samples with a total of 404 samples. Grey boxes correspond to healthy samples, with a total of 28 samples. * $p \leq 0.05$.

Journal of Cell Science • Supplementary information

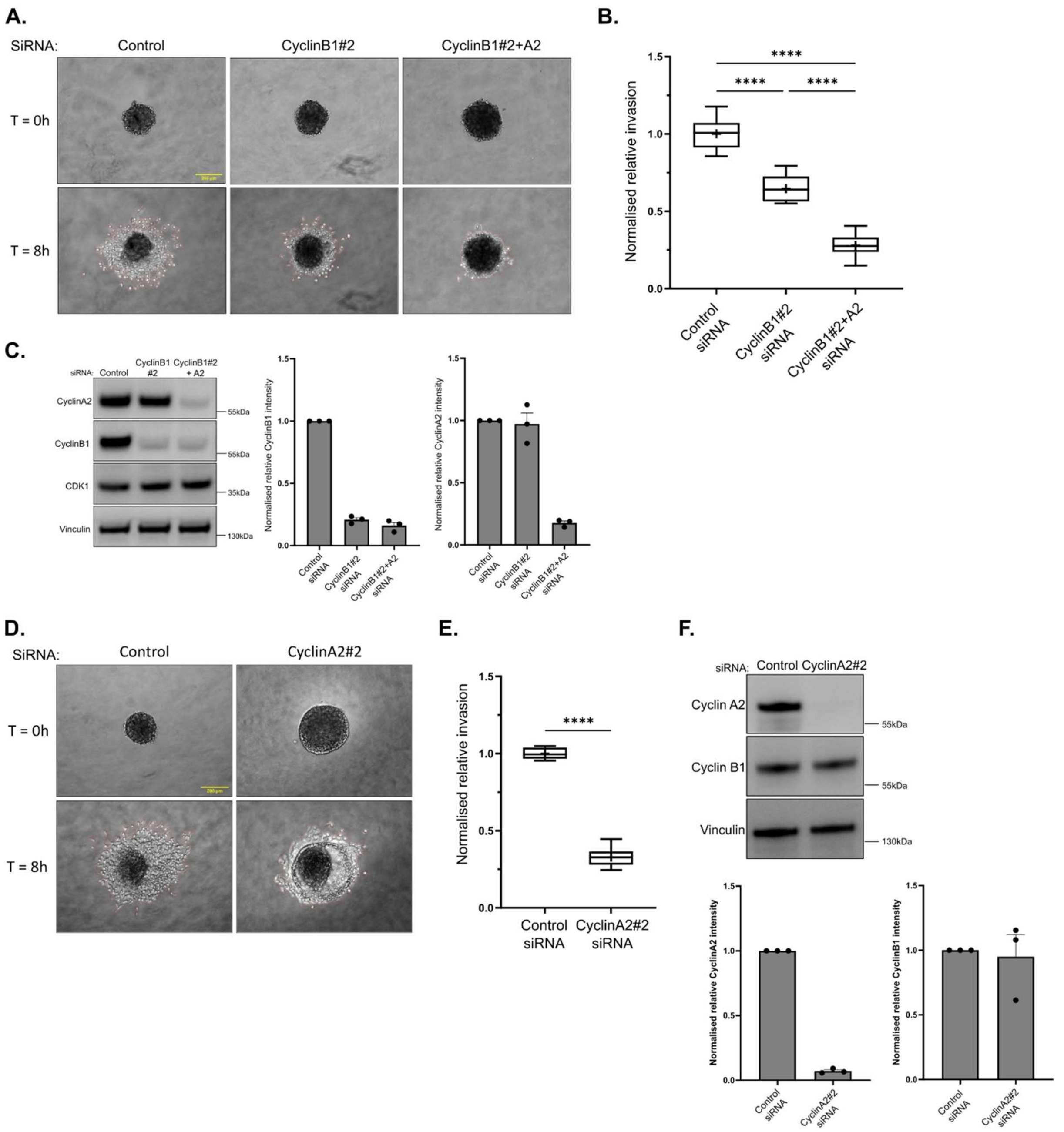

Fig. S6. Knockdown of cyclin A2 and B1 using a second siRNA oligo perturbs T24 invasion in collagen gels. **A** T24 spheroid invasion assay showing T24 spheroids generated from control, cyclinB1 and cyclin B1 +A2 knockdown cells using a cyclinB1 siRNA distinct from the one used in Figure 5, seeded into 2.5mg/ml collagen gels at 0 hour and 8 hour timepoints **B.** Quantification of invasion area of cyclinB1 and cyclin A2 +B1 knockdown T24 spheroids relative to control siRNA spheroids. 9 spheroids per condition across 3 repeats was used for analysis. **C.** Western blot shows protein levels of cyclinA2, cyclinB1, CDK1 and vinculin in control siRNA, cyclinB1#2 siRNA and cyclinB1#2+A2 siRNA T24 cells that were used for spheroid generation. Graphs show quantification of cyclinA2 and cyclinB1 levels from 3 independent experiments. **D.** T24 spheroid invasion assay showing T24 spheroids generated from cyclinA2 knockdown T24 cells using an siRNA distinct from Figure 5 and A-C here, seeded into 2.5mg/ml collagen gels at 0 hour and 8 hour timepoints **E.** Quantification of invasion area of cyclinA2#2 knockdown spheroids relative to control siRNA treated spheroids. 9 spheroids per condition across 3 repeats was used for analysis. **F.** Western blot shows protein levels of cyclinA2, cyclinB1 and vinculin in control siRNA and cyclinA2#2 siRNA T24 cells that were used for spheroid generation. Graphs show quantification of cyclinA2 and cyclinB1 levels from 3 independent experiments.

Journal of Cell Science • Supplementary information

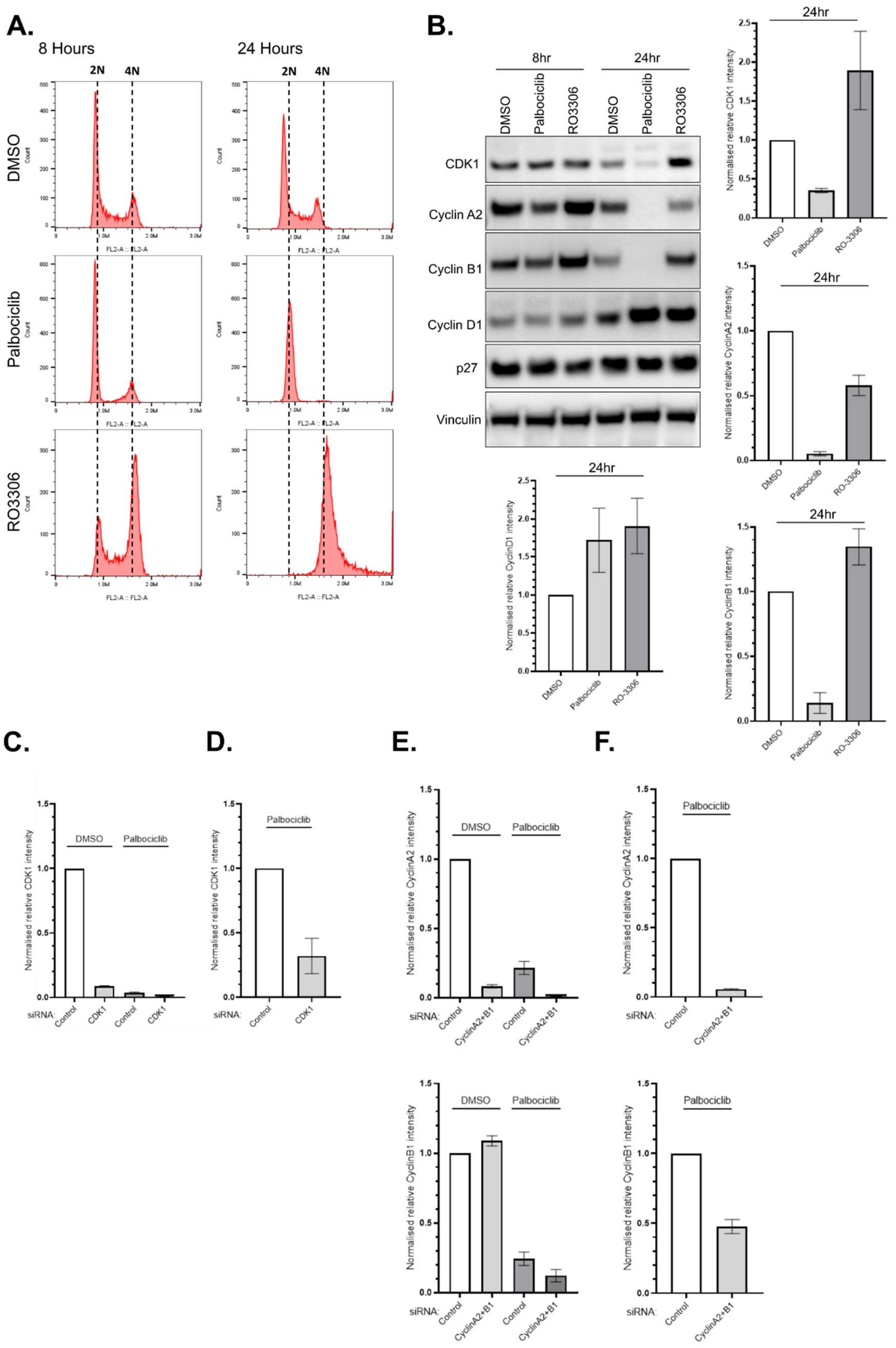

Journal of Cell Science • Supplementary information

Fig. S7. Treatment with palbociclib for 24 hours leads to a reduction in CDK1, cyclinA2 and cyclinB1. Knockdown of CDK1 and cyclins leads to a further reduction in invasion in palbociclib treated cells A. Flow cytometry analysis showing cell cycle profiles of T24 cells following 8h and 24h treatment with DMSO, Palbociclib or RO-3306 using propidium iodide-stained cells. **B.** Western blot showing CDK1, cyclinA2, cyclinB1, cyclinD1, p27 and Vinculin (for loading) protein levels for T24 cells following 8h and 24h treatment with DMSO, Palbociclib or RO-3306. Quantification of band intensities for indicated proteins from 3 independent experiments shown in graphs **C.** Quantification of western blots from Figure 7. highlighting CDK1 protein levels of CDK1 knockdown T24 cells treated with DMSO or Palbociclib relative to DMSO-treated control siRNA cells. Error bars show SEM **D.** Quantification of western blot from Figure 7. highlighting relative CDK1 protein levels of palbociclib treated cells only. Error bars show SEM from 3 independent experiments **E.** Quantification of western blots from Figure 7. highlighting cyclinA2 and cyclnB1 protein levels of A2+B1 knockdown T24 cells treated with DMSO or Palbociclib relative to DMSO-treated control siRNA cells. Error bars show SEM **F.** Quantification of western blot from Figure 7. highlighting relative cyclinA2 and cyclinB1 protein levels of palbociclib treated cells only. Error bars show SEM from 3 independent experiments.

Journal of Cell Science • Supplementary information

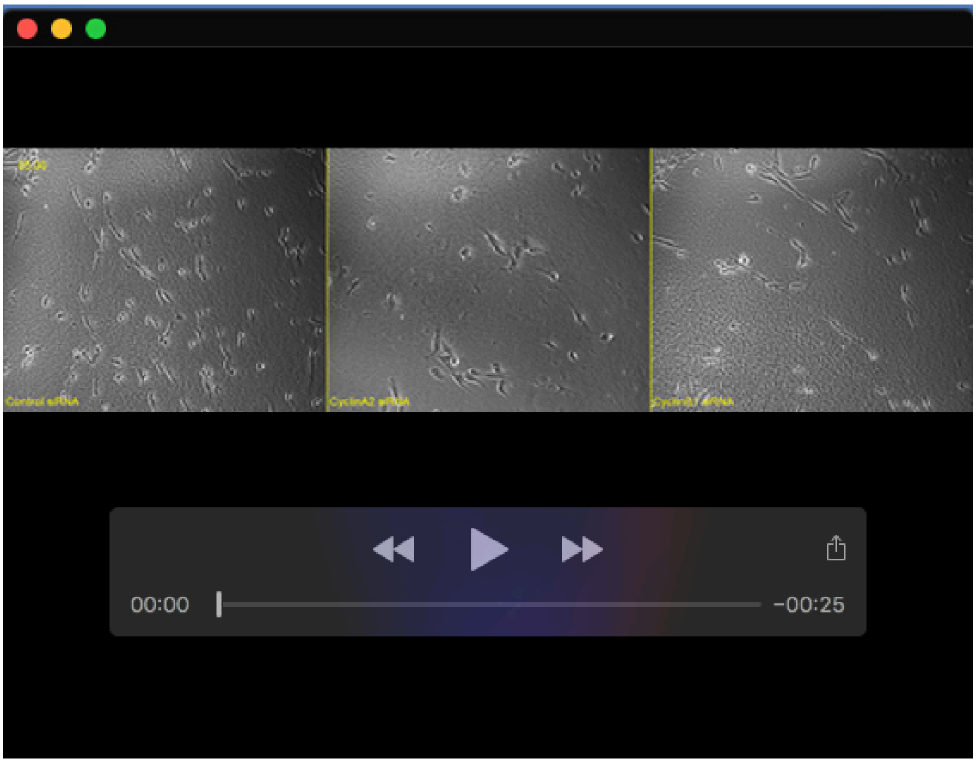

Movie 1. Timelapse movie of control (left), cyclinA2 (centre) and cyclinB1 (right) siRNA A2780 cells moving in CDM over 16 hour timelapse.

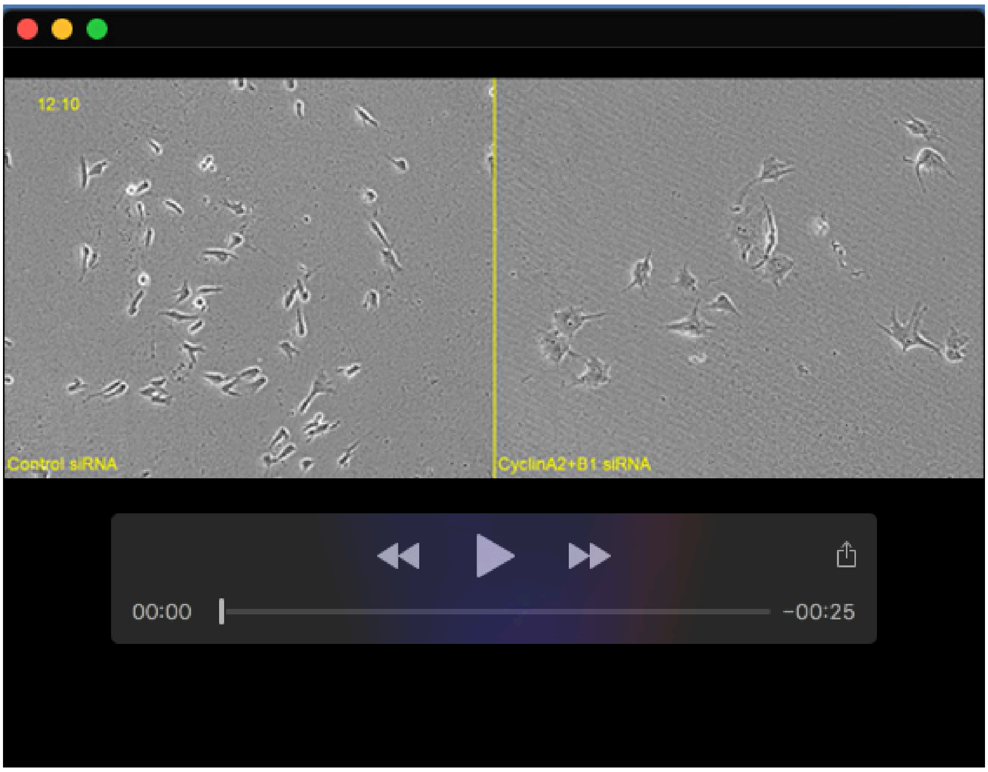

Movie 2. Timelapse movie of control (left), and cyclinA2+B1 (right) siRNA A2780 cells moving in CDM over 60 hour timelapse.

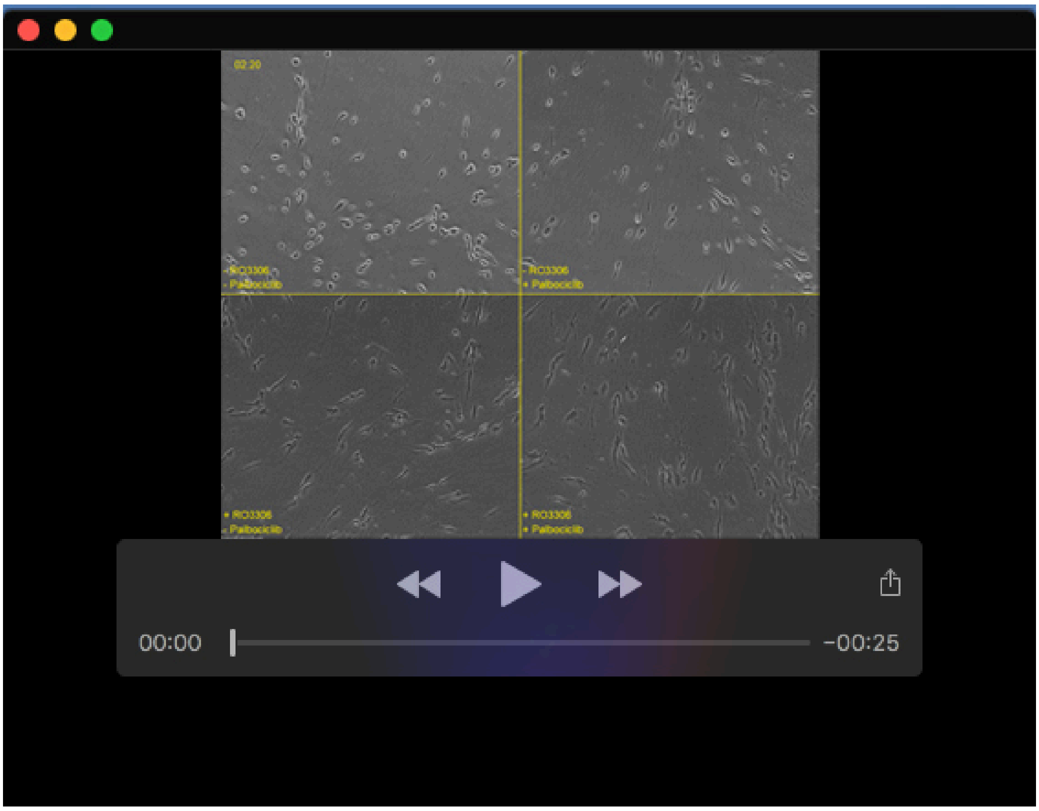

Movie 3. Timelapse movie of cells moving in CDM over 16 hour timelapse treated with RO- 3306 and Palbociclib as indicated (DMSO used for control)
